# Abnormal adipose tissue-derived microbes drive metabolic disorder and exacerbate postnatal growth retardation in piglet

**DOI:** 10.1093/lifemeta/load052

**Published:** 2024-01-17

**Authors:** Tongxing Song, Ming Qi, Yucheng Zhu, Nan Wang, Zhibo Liu, Na Li, Jiacheng Yang, Yanxu Han, Jing Wang, Shiyu Tao, Zhuqing Ren, Yulong Yin, Jinshui Zheng, Bie Tan

**Affiliations:** College of Animal Science and Technology, Huazhong Agricultural University, Wuhan, Hubei 430070, China; College of Animal Science and Technology, Hunan Agricultural University, Changsha, Hunan 410000, China; Laboratory of Animal Nutritional Physiology and Metabolic Process, CAS Key Laboratory of Agro-ecological Processes in Subtropical Region, National Engineering Laboratory for Pollution Control and Waste Utilization in Livestock and Poultry Production, Institute of Subtropical Agriculture, Chinese Academy of Sciences, Changsha, Hunan 410125, China; College of Animal Science and Technology, Huazhong Agricultural University, Wuhan, Hubei 430070, China; College of Animal Science and Technology, Hunan Agricultural University, Changsha, Hunan 410000, China; Laboratory of Animal Nutritional Physiology and Metabolic Process, CAS Key Laboratory of Agro-ecological Processes in Subtropical Region, National Engineering Laboratory for Pollution Control and Waste Utilization in Livestock and Poultry Production, Institute of Subtropical Agriculture, Chinese Academy of Sciences, Changsha, Hunan 410125, China; College of Animal Science and Technology, Huazhong Agricultural University, Wuhan, Hubei 430070, China; State Key Laboratory of Agricultural Microbiology, Huazhong Agricultural University, Wuhan, Hubei 430070, China; Hubei Key Laboratory of Agricultural Bioinformatics, Huazhong Agricultural University, Wuhan, Hubei 430070, China; College of Animal Science and Technology, Huazhong Agricultural University, Wuhan, Hubei 430070, China; College of Animal Science and Technology, Huazhong Agricultural University, Wuhan, Hubei 430070, China; College of Animal Science and Technology, Hunan Agricultural University, Changsha, Hunan 410000, China; Laboratory of Animal Nutritional Physiology and Metabolic Process, CAS Key Laboratory of Agro-ecological Processes in Subtropical Region, National Engineering Laboratory for Pollution Control and Waste Utilization in Livestock and Poultry Production, Institute of Subtropical Agriculture, Chinese Academy of Sciences, Changsha, Hunan 410125, China; College of Animal Science and Technology, Huazhong Agricultural University, Wuhan, Hubei 430070, China; College of Animal Science and Technology, Huazhong Agricultural University, Wuhan, Hubei 430070, China; College of Animal Science and Technology, Hunan Agricultural University, Changsha, Hunan 410000, China; Laboratory of Animal Nutritional Physiology and Metabolic Process, CAS Key Laboratory of Agro-ecological Processes in Subtropical Region, National Engineering Laboratory for Pollution Control and Waste Utilization in Livestock and Poultry Production, Institute of Subtropical Agriculture, Chinese Academy of Sciences, Changsha, Hunan 410125, China; State Key Laboratory of Agricultural Microbiology, Huazhong Agricultural University, Wuhan, Hubei 430070, China; Hubei Key Laboratory of Agricultural Bioinformatics, Huazhong Agricultural University, Wuhan, Hubei 430070, China; College of Animal Science and Technology, Hunan Agricultural University, Changsha, Hunan 410000, China; Laboratory of Animal Nutritional Physiology and Metabolic Process, CAS Key Laboratory of Agro-ecological Processes in Subtropical Region, National Engineering Laboratory for Pollution Control and Waste Utilization in Livestock and Poultry Production, Institute of Subtropical Agriculture, Chinese Academy of Sciences, Changsha, Hunan 410125, China

**Keywords:** adipose tissue, microbe, metabolic disorder, postnatal growth retardation, piglet

## Abstract

Postnatal growth retardation (PGR) frequently occurs during early postnatal development of piglets and induces high mortality. To date, the mechanism of PGR remains poorly understood. Adipose tissue-derived microbes have been documented to be associated with several disorders of metabolism and body growth. However, the connection between microbial disturbance of adipose tissue and pig PGR remains unclear. Here, we investigated piglets with PGR and found that the adipose tissue of PGR piglets was characterized by metabolism impairment, adipose abnormality, and specific enrichment of culturable bacteria from *Proteobacteria*. Gavage of *Sphingomonas paucimobilis*, a species of *Sphingomonas* genus from the *alphaproteobacteria*, induced PGR in piglets. Moreover, this bacterium could also lead to metabolic disorders and susceptibility to acute stress, resulting in weight loss in mice. Mechanistically, multi-omics analysis indicated the changes in lipid metabolism as a response of adipose tissue to abnormal microbial composition. Further experimental tests proved that one of the altered lipids phosphatidylethanolamines could rescue the metabolism disorder and growth retardation, thereby suppressing the amount of *Sphingomonas* in the adipose tissue. Together, these results highlight that the microbe–host crosstalk may regulate the metabolic function of adipose tissue in response to PGR.

## Introduction

As one of the most important livestock species in the world, pigs are an important source of meat, as well as serve as a good bio-model for research on human diseases [1, 2]. During the early life of pigs, postnatal growth retardation (PGR) with low growth rate and lifelong deficits in growth and development is associated with metabolic disorders, leading to high mortality in pig production [2, 3]. Moreover, PGR also increases feed disappearance by 10%–30% and production cost [2, 3]. In humans, millions of children not only fail to achieve adequate bone development and immune system but also have deficits in whole-body metabolic homeostasis, resulting in perturbed linear growth and low body weight (BW) [4]. However, the mechanism of PGR remains poorly understood. Adipose tissue is a well-known organ for both energy storage and endocrinal regulation and functions by secreting adipokines into the circulation system and modulating glucose uptake and insulin resistance, thereby regulating the whole-body development in early life [5–7]. It has been found that child stunting is usually related to changes in the morphology and function of adipose tissue [8–10]. For example, under acute malnutrition, signals from adipose tissue could suppress the metabolic activity of the body and promote energy mobilization [10]. However, the metabolic role of adipose tissue in PGR remains largely unknown.

Few studies have indicated that gut and environmental microbes can be translocated into the adipose tissue and play a regulatory role in whole-body metabolism [11–15]. Enrichment of microbes in the adipose tissue from individuals has been reported to be associated with metabolic disorders such as chronic inflammation, obesity, diabetes, and creeping fat from Crohn’s disease [11–13]. Interestingly, viable microbes in human adipose tissue were found to polarize macrophages and promote adipogenesis, thereafter resulting in the formation of creeping fat [15]. Besides, the translocated microbes were found to affect the transcriptome and metabolome of human mesenteric adipose tissue to exacerbate the Crohn’s disease in a mouse model [14]. In both obese individuals with and without healthy metabolism, the main bacteria derived from adipose tissue belong to the phylum of *Proteobacteria*, a phylum causing high motility [12, 14], which might explain the presence of microbes in several organs and blood [16]. Nevertheless, some critical questions remain to be answered, including whether certain adipose tissue-derived microbe is associated with chronic malnutrition and the role of microbe in metabolic function of adipose tissue in PGR.

Animal models are important and essential tools for translational and clinical research on human growth, development, and diseases [4, 17]. Compared with other primate and lab animal models such as mice and rats, pigs have many advantages in anatomy and physiology, making them an ideal model for translational biomedical research on metabolic diseases [1, 18]. Several piglet models have been established for studies of diseases during early life, including bacterial infection [19], diarrhea [20], and malnutrition [21]. To further develop potential interventions for child patients [22], piglet models are preferably used in translational and pre-clinical research on early-life malnutrition [23, 24]. Recently, an intergenerational pig model of diet restriction mimicking undernutrition children was employed to clarify the regulatory role of gut microbes in ponderal growth [25]. Besides, models for metabolic syndromes, including chronic inflammation, insulin resistance, and diabetes, have also been successfully established in pigs [26, 27]. Previously, researchers have employed piglets as models to understand the mechanisms and therapeutic methods of PGR and intrauterine growth retardation in humans [20, 21, 25, 28, 29]. Therefore, pigs may serve as an extensive and vital large animal model due to the advantages such as consistent genomes and growth phenotypes, large litters, and highly efficient reproduction.

In the present study, we established a piglet PGR model and analyzed its growth and metabolic characters. A well-defined microbe derived from the adipose tissue was gavaged to reestablish the phenotype of metabolic disorder and growth retardation in pigs. In addition, the effect of phosphatidylethanolamine (PE), a key metabolite likely secreted from the adipose tissue in response to microbes, on the growth retardation was investigated.

## Results

### Characteristics of a piglet postnatal growth retardation model

**Figure 1:**
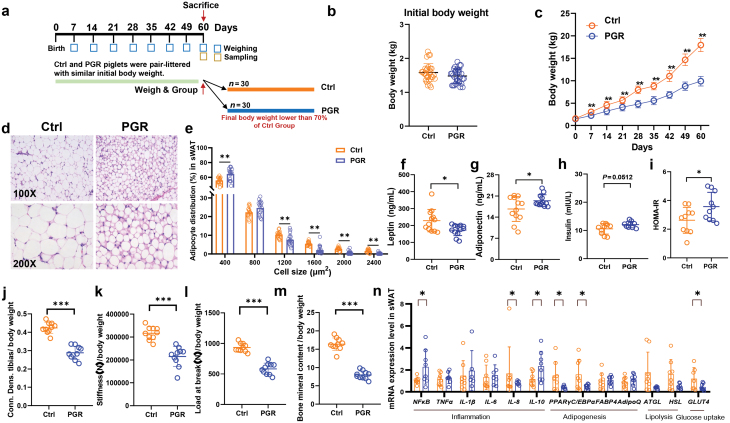
Differences in adipose tissue metabolic function and histological structure between the Ctrl and PGR piglets. (a) Diagram of the experiment design. (b) Initial BW. (c) Cumulative BW of the Ctrl and PGR piglets. (d and e) Histological score of sWAT in the Ctrl and PGR piglets. The magnification is 100 (upper) and 200 (under). (f–i) Levels of leptin (f), adiponectin (g), insulin (h), and HOMA-IR (i) in the Ctrl and PGR piglets. (j–m) Conn. Dens. tibias (j), stiffness (k), load at break (l), and bone mineral content (M) of the Ctrl and PGR piglets. (n) mRNA levels of genes related to inflammation (*TNFα*, *IL-1β*, and *IL-10*), adipogenesis (*PPARγ, C/EBPα, AdipoQ*, and *FABP4*), lipolysis (*HSL* and *ATGL*), and glucose uptake (*GLUT4*) in sWAT were assessed by qPCR. The statistical significance is denoted as: ^*^P < 0.05, ^**^P < 0.01, ^***^P < 0.001

To investigate the mechanism of PGR, we generated 30 litters of newborn piglets from 30 sows, with a total of 370 newborns (average 12 newborns per litter) of the same genetic background (Fig. 1a). PGR piglets were selected with the following criteria: (i) birth weight higher than 1.1 kg, with no difference in birth weight relative to control (Ctrl) piglets (Fig. 1b); (ii) BW less than 70% average BW of Ctrl piglets on Day 60; (iii) no obvious characteristics of injury [30]. All the piglets were raised together, and one Ctrl piglet and one PGR piglet were randomly selected in the same litter and sacrificed on Day 60 (Fig. 1c). We carefully collected samples of fat from different deposits including subcutaneous white adipose tissue from the back (sWAT) and abdominal white adipose tissue (aWAT), which play different metabolic roles [31], as well as whole blood and liver under sterile conditions. The samples were assessed with different omics datasets, including metagenomics, transcriptomics, and metabolomics.

The BW of PGR piglets was remarkably smaller than that of the Ctrl group from Day 7 after birth (Fig. 1c). Consistent with the final BW, the PGR group exhibited a lower weight gain in the whole test period (Supplementary Fig. S1a). To determine whether the PGR group was clinically relevant to growth retardation, the bone morphology and density were compared between the Ctrl and PGR groups by dual-energy X-ray absorptiometry. As a result, the thighbones of the PGR group showed shorter length (Supplementary Fig. S1b) and lower bone strength and density (Fig. 1j–m), demonstrating high similarities to the phenotype of growth retardation [32].

To explore the impact of adipose tissue on PGR, a histology analysis with hematoxylin and eosin (H&E) staining on the adipose tissue was performed. The results revealed significant differences in adipose morphology between the Ctrl and PGR groups: the PGR group had a large number of smaller adipocytes (Fig. 1d and e). Since the metabolic function of adipose tissue in homeostasis is closely related to its morphology [31], it could be speculated that metabolic disorders occurred in the PGR group. To identify the metabolic distinction between the two groups, we further compared the adipokine levels in serum [6]. The results demonstrated that the PGR group had a significantly lower level of leptin while a significantly higher level of adiponectin than the Ctrl group (Fig. 1f and g). Compared with the Ctrl group, the PGR group showed an increasing tendency (*P* = 0.0512) in fasting insulin level (Fig. 1h), but there was no difference in the level of fasting glucose (Supplementary Fig. S1c). The PGR group developed insulin resistance as indicated by the increased HOMA-IR (homeostasis model assessment insulin resistance) index calculated from fasting glucose and insulin [33] (Fig. 1i). Furthermore, the key genes related to adipogenesis and glucose uptake in adipose tissue were down-regulated in the sWAT of the PGR group (Fig. 1n). In addition, nearly all genes related to inflammation showed no difference between the two groups, except for an increase in nuclear factor-κB (*NF*κ*B*) and interleukin 10 (*IL-10*) and a decrease in *IL-8* in the sWAT of the PGR group (Fig. 1n). These results suggested that the abnormal morphology and metabolic dysfunction of the adipose tissue are associated with PGR, indicating the potential role of adipose tissue in growth retardation.

### The relative abundance of bacteria from the phylum *Proteobacteria* in adipose tissue is negatively associated with body weight of piglets

**Figure 2:**
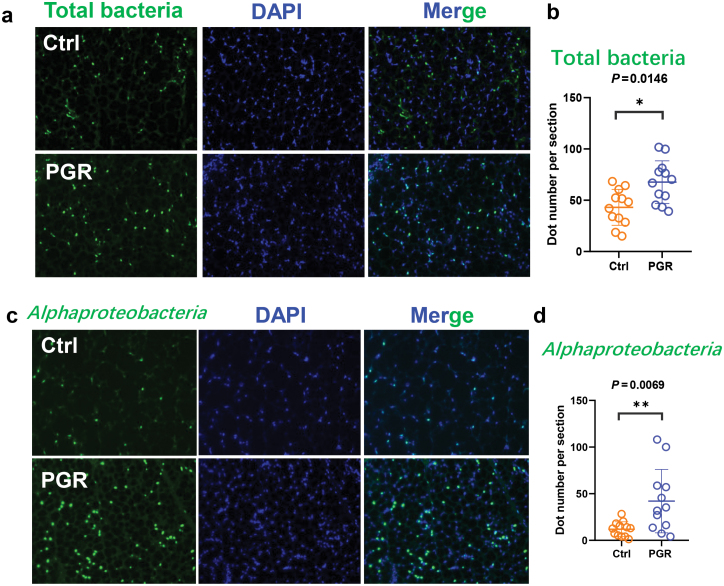
CARD-FISH analysis for piglet adipose tissue. (a) CARD-FISH on adipose tissue section from the Ctrl and PGR piglets: total bacteria and DAPI staining. Magnification is 200. (b) Positive dot number per section for total bacteria by CARD-FISH. (c) CARD-FISH on adipose tissue section from the Ctrl and PGR piglets: *Alphaproteobacteria* (green) and DAPI staining (blue). Magnification is 200. (d) Positive dot number per section for *Alphaproteobacteria* by CARD-FISH. The statistical significance is denoted as: ^*^P < 0.05, ^**^P < 0.01.

Previous studies have demonstrated that the translocation of certain bacterial members from the gut to adipose tissue is linked to metabolic dysfunction [12]. In the above experiment, we observed abnormal morphology of adipose tissue and metabolic dysfunction in PGR piglets. It could be hypothesized that microbe from the adipose tissue may be associated with PGR by affecting whole-body metabolic homeostasis. To test this hypothesis, catalyzed reporter deposition fluorescence *in situ* hybridization (CARD-FISH) test was employed to detect the number of microbes in adipose tissue. To reduce contamination from environment during processing and exclude false-positive findings of CARD-FISH, adipose tissue collected from germ-free mice was sectioned for CARD-FISH analysis. As expected, no positive signals were found, which is consistent with the results detected without probes (negative control), indicating the reliability of this method (Supplementary Fig. S2). Subsequently, a significant increase in the numbers of both total bacteria and *alphaproteobacteria* was observed in the adipose tissue of PGR piglets (Fig. 2a–d). Taken together, these results revealed the presence of microbes in the adipose tissue of the PGR group, which may be related to growth retardation.

### A defined intra-tissue genus *Sphingomonas* in *alphaproteobacteria* class is enriched in adipose tissue of postnatal growth retardation piglets

Tissue-resident microbiota samples are easily contaminated by host and environmental noise [13, 34]. Thus, it is necessary to carefully check the contamination from environment and host genome. As shown in Supplementary Fig. S3, four groups were set up for amplifying V3–V4 region of bacterial 16S rRNA gene to detect the contamination from the DNA extraction kit, environment, and host genome. As expected, no band was found in these three groups and a clear band was observed in the positive group with bacterial DNA as a template (Supplementary Fig. S3a), providing evidence to exclude contamination in this study. Next, PCR amplicons with a clear band were purified and quantified for further 16S library construction (Supplementary Fig. S3b). Thus, the contamination from host genome could be excluded.

**Figure 3:**
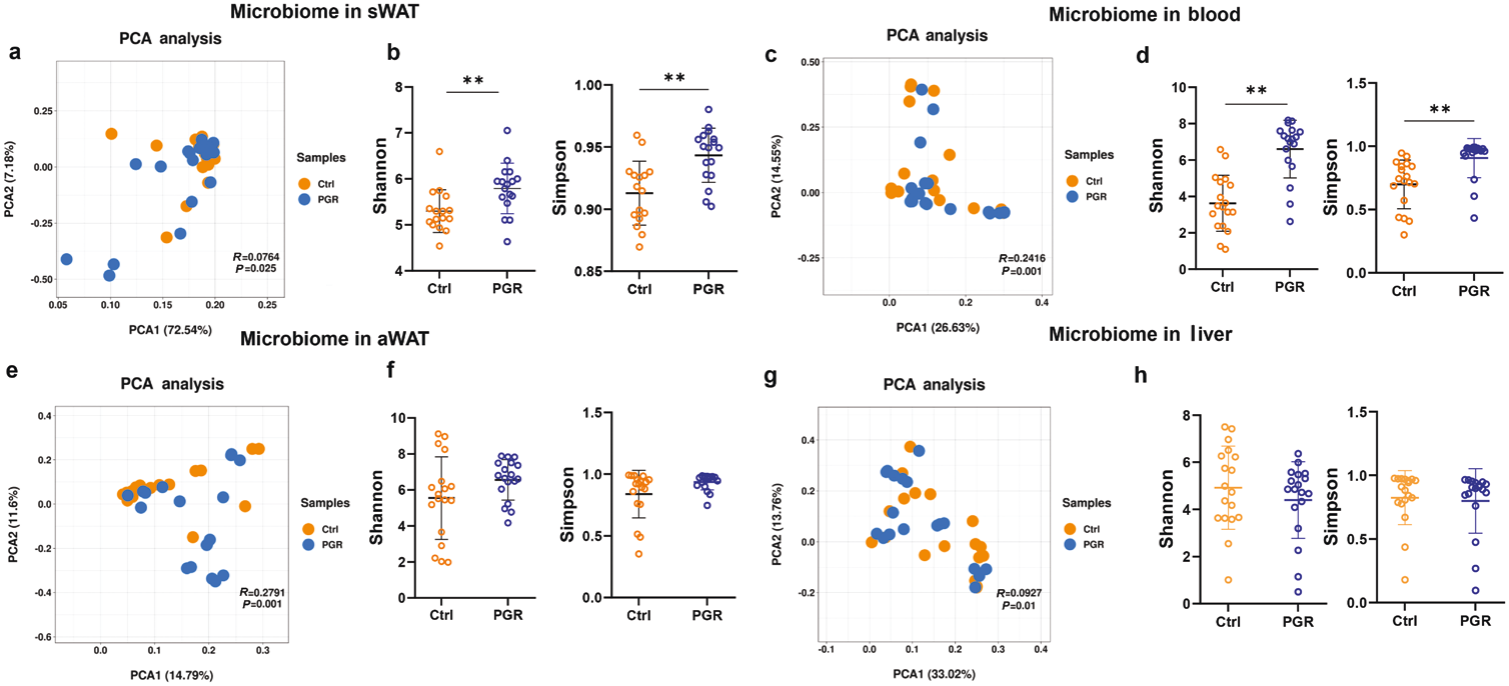
Microbiome signatures of sWAT, aWAT, blood, and liver in the Ctrl group versus the PGR group. (a) Separation of sWAT microbiome between the Ctrl and PGR piglets revealed by PCA (Adonis *P* = 0.025). (b) Shannon index and Simpson index in sWAT. (c) Separation of blood microbiome between the PGR and Ctrl piglets revealed by PCA (Adonis *P* = 0.001). (d) Shannon index and Simpson index in blood. (e) Separation of aWAT microbiome between the PGR and Ctrl piglets revealed by PCA (Adonis *P* = 0.001). (f) Shannon index and Simpson index in aWAT. (g) Separation of liver microbiome between the PGR and Ctrl piglets revealed by PCA (Adonis *P *= 0.01). (h) Shannon index and Simpson index in liver. The statistical significance is denoted as ^*^P < 0.05, ^**^P < 0.01.

To verify the specific microbial signature in the adipose tissue of PGR piglets, 16S rRNA gene sequencing of sWAT, aWAT, liver, and blood was performed to examine the differences in intra-tissue microbes between the Ctrl and PGR groups (Fig. 3; Supplementary Fig. S3b). Interestingly, significant variations of bacterial community were found in different tissues by β-diversity analysis, suggesting that different tissues showed specific characteristics of bacterial community. This might also indicate low contamination from the environment in these tissues (Supplementary Fig. S4). In addition, the bacterial community structure of the microbes in sWAT was small, but there were significant differences between the Ctrl and PGR groups (Fig. 3a; Supplementary Fig. S5a). Compared with that of the Ctrl group, the microbial α-diversity of the PGR group was higher in the sWAT and blood but had no difference in the liver and aWAT (Fig. 3a–h). Bacterial community composition analysis at the phylum level demonstrated that *Firmicutes* and *Proteobacteria* were the dominant phyla in the adipose tissue (Supplementary Fig. S5a–d), relative to the other top five bacteria. Interestingly, we further analyzed the *Proteobacteria* with high motility and *alphaproteobacteria* showed a significant increase in sWAT (Supplementary Fig. S5e). To further clarify the microbial differences, we compared the bacteria at the genus level. As shown in Supplementary Fig. S6a and b, a number of genera were significantly upregulated in sWAT and aWAT. Because of large variations within groups, there was no remarkable difference in the liver and blood, indicating main bacterial translocation in adipose tissue.

**Figure 4:**
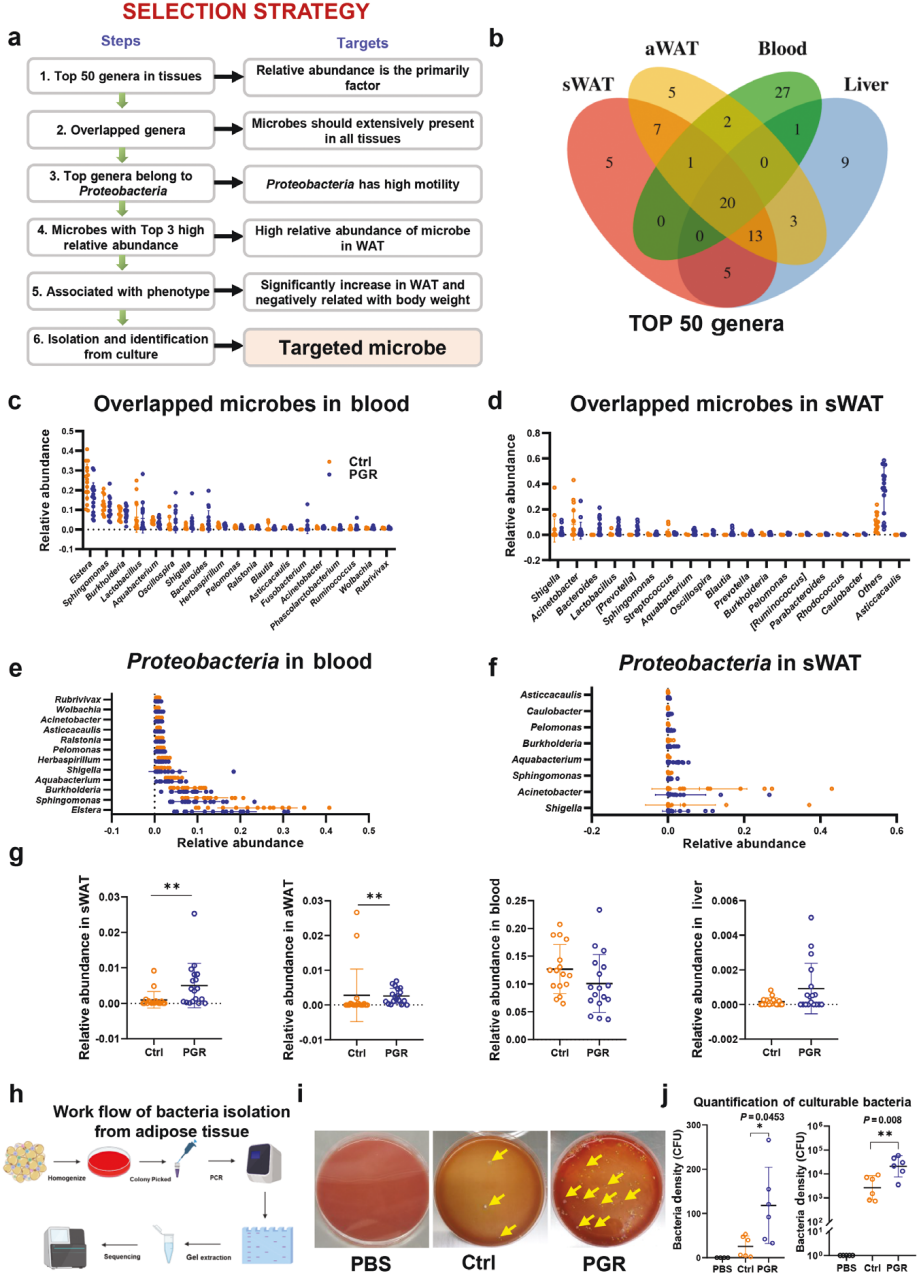
Identification of a genus in *alphaproteobacteria* class (*Sphingomonas*) in porcine adipose tissue. (a) Diagram of selection strategy for a targeted microbe. (b) Top 50 overlapped genera in the four tissues. (c and d) Top 20 overlapped genera in the blood (c) and sWAT (d). (e and f) Relative abundance of genera from *Proteobacteria* in the blood (e) and sWAT (f). (g) Relative abundance of *Sphingomonas* in sWAT, aWAT, blood, and liver. (h) Workflow of bacteria isolation from adipose tissue. (i) Representative culture plate in the Ctrl and PGR groups. PBS is the negative control. Yellow arrows show the colonies. (j) Quantification of cultivated bacteria in the Ctrl and PGR groups. The statistical significance is denoted as ^*^P < 0.05, ^**^P < 0.01.

Next, to detect the key microbes at the low taxonomic level responsible for the difference between the PGR and Ctrl groups, a selection strategy was designed for identifying targeted microbes (Fig. 4a). We first compared the top 50 genera with the highest relative abundance from sWAT, aWAT, liver, and blood (Fig. 4b). Then, a total of 20 genera were overlapped by four tissues, implying the potential translocation of these bacteria in the body (Fig. 4c). The relative abundance of overlapped 20 genera from blood and sWAT was determined (Fig. 4c and d; Supplementary Fig. S6c and d). We subsequently focused on genera in the *alphaproteobacteria* class and found 12 genera in the blood and 8 genera in sWAT, aWAT, and liver (Fig. 4e and f; Supplementary Fig. S6e and f). Although the relative abundance of microbes was low in both the blood and sWAT, we focused on the top three microbes with high relative abundance. As shown in Fig. 4g, we compared the relative abundance of these three microbes between the Ctrl and PGR groups in adipose tissues. Interestingly, only *Sphingomonas* derived from the adipose tissue significantly increased in the PGR group, while that derived from the blood and liver showed no change (Fig. 4g; Supplementary Fig. S7a–d). Another microbe *Shigella* decreased in the adipose tissue of the PGR group (Supplementary Fig. S7a and b). Then, we tried to culture bacteria belonging to *Sphingomonas* from adipose tissue. Based on the work flow shown in Fig. 4h, we cultured and quantified the adipose tissue-resident bacteria by culturing the homogenized adipose tissue on Columbia blood agar base plate [34]. The results showed a median of 25 colony-forming units (CFU) for adipose tissue for the Ctrl group and 118 CFU for the PGR group (Fig.4i and j). Subsequently, the isolated bacteria were detected by specific PCR primers for *Sphingomonas* and clear bands were observed, suggesting that the culturable bacterium may belong to *Sphingomonas* (Supplementary Fig. S8). Notably, this culturable bacterium can also be successfully amplified by primers for *Sphingomonas paucimobilis* (*S. paucimobilis*, ATCC 29837), which acts as a standard bacterium from *Sphingomonas* [35, 36]. Furthermore, whole genome sequence (WGS) was performed for analyzing the similarity of cultivated and commercial *S. paucimobilis*. As shown in Supplementary Fig. S9a and b, the average nucleotide identity (ANI) values were 99.98%, which is considered to be the most relevant comparative parameter used for bacterial species delineation, suggesting high similarity between the two stains. Then, comparative genomics revealed that there were 3849 operational gene units that comprise the conserved core of *S. paucimobilis* genome, and 193 unique genes were detected. The Kyoto Encyclopedia of Genes and Genomes (KEGG) and the Clusters Of Orthologous Genes (COG) function analysis indicated that *S. paucimobilis* may be related to lipid metabolism and energy metabolism (Supplementary Fig. S9c and d). Together, these data indicated a close association between microbe and growth retardation, suggesting that the retardation of piglet growth may be related to adipose tissue-derived microbe, particularly those from the *Sphingomonas* genus.

### Gavage of *Sphingomonas* leads to postnatal growth retardation in piglets

**Figure 5:**
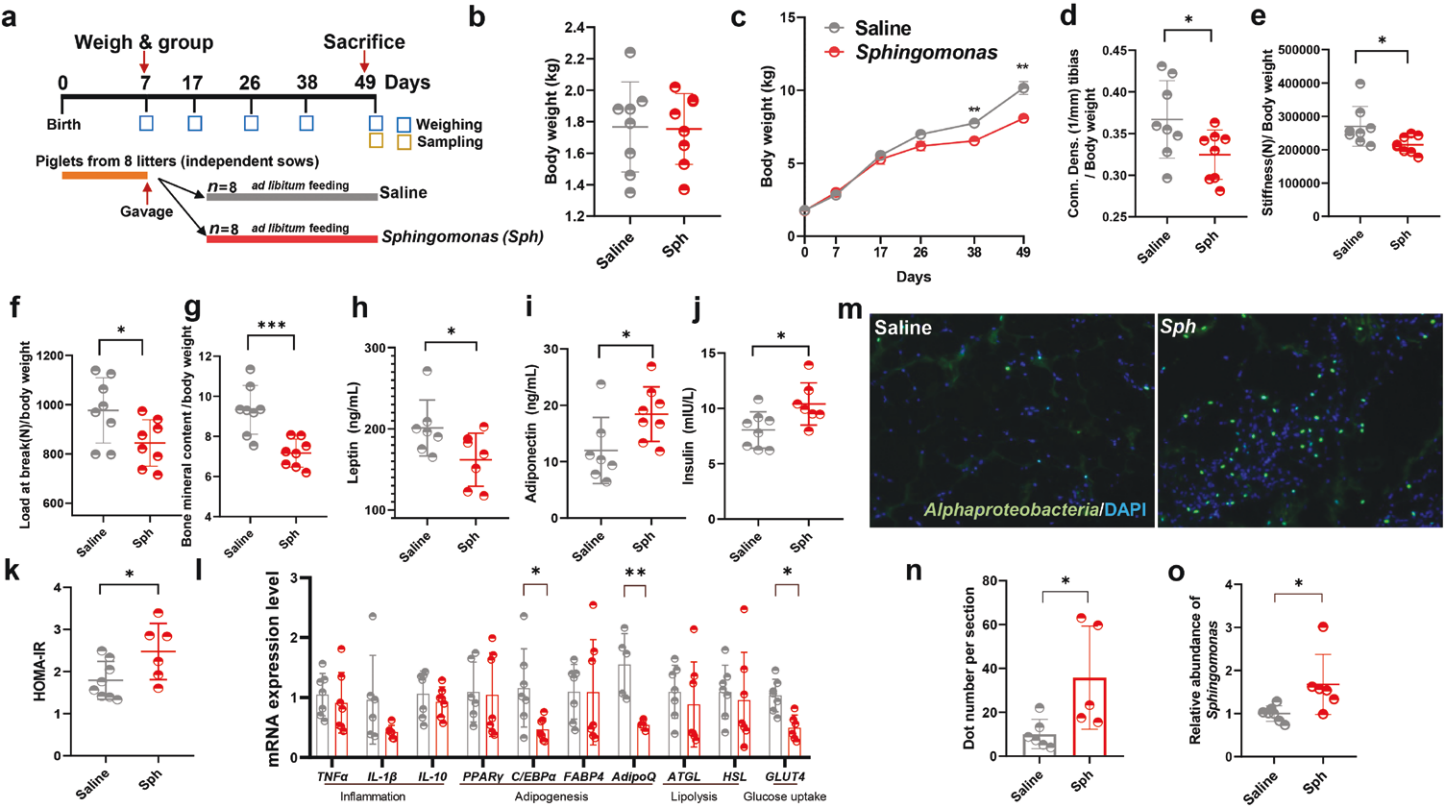
Gavage of *Sphingomonas* induces the formation of PGR. (a) Diagram of the experiment design. (b and c) Initial BW (b) and cumulative BW (c) of Saline and *Sphingomonas-*gaveged piglets. (d–g) Index of bone morphology: Conn. Dens. tibias (d), stiffness (e), load at break (f), and bone mineral content (g) of Saline and *Sphingomonas*-gavaged groups. (h–k) Levels of leptin (h), adiponectin (i), insulin (j), and HOMA-IR (k) in Saline and *Sphingomonas* piglets. (l) mRNA levels of genes related to inflammation (*TNFα*, *IL-1β*, and *IL-10*), adipogenesis (*PPARγ*, *C/EBPα*, *AdipoQ*, and *FABP4*), lipolysis (*HSL* and *ATGL*), and glucose uptake (*GLUT4*) in sWAT assessed by qPCR. (m) CARD-FISH on adipose tissue section from Saline and *Sphingomonas* piglets: *Alphaproteobacteria* and DAPI staining. Magnification is 100. (n) Positive dot number per section for *Alphaproteobacteria* by CARD-FISH. (o) Relative abundance of *Sphingomonas* detected by qPCR. The statistical significance is denoted as: ^*^P < 0.05, ^**^P < 0.01, ^***^P < 0.001.

To test whether bacteria from the *Sphingomonas* genus play a role in inducing PGR in piglets, one isolate of *Sphingomonas*, *S. paucimobilis* (ATCC 29837), was cultured for further study. Piglets were daily gavaged for 6 weeks with freshly prepared live *S. paucimobilis*, which was washed and diluted by saline (1 × 10^9^ live bacteria/day/piglet; 6 weeks from Day 7 after birth to Day 49) (Fig. 5a). *S. paucimobilis*-colonized piglets (*Sphingomonas* group) exhibited a comparable weight gain to the PGR piglets, but significantly lower weight gain relative to the Saline group (Fig. 5b and c; Supplementary Fig. S10a and b). Consistently, the *Sphingomonas* group showed lower bone strength and bone density (Fig. 5d–g) and thighbone length (Supplementary Fig. S10c). To further test whether the *Sphingomonas* group had similar phenotypes in metabolism of adipose tissue to the PGR group, adipokines were measured in sWAT. The same changing pattern of leptin and adiponectin was found in the *Sphingomonas* group and the PGR group (Fig. 5h and i; Fig. 1f and g). Also, the *Sphingomonas* group had similar fasting insulin and glucose levels to the PGR group, as well as same insulin resistance as indicated by the HOMA-IR index (Fig. 5j and k; Supplementary Fig. S10d). In addition, the administration of *S. paucimobilis* significantly downregulated the expression of *CEBPα* and *Glut4* (Fig. 5l). To further confirm the translocation of *S. paucimobilis* in the adipose tissue, we performed CARD-FISH using *alphaproteobacteria*-specific probes under sterile conditions and quantitative real-time PCR (qPCR) using *Sphingomonas*-specific primers. The results showed a higher abundance of *Sphingomonas* in sWAT in the *Sphingomonas* group (Fig. 5m–o). These findings suggested that *Sphingomonas* might be a potential contributor to the formation of PGR in piglets.

### *S. paucimobilis-*colonized mice exhibit metabolic disorders and body weight loss under acute stress

**Figure 6:**
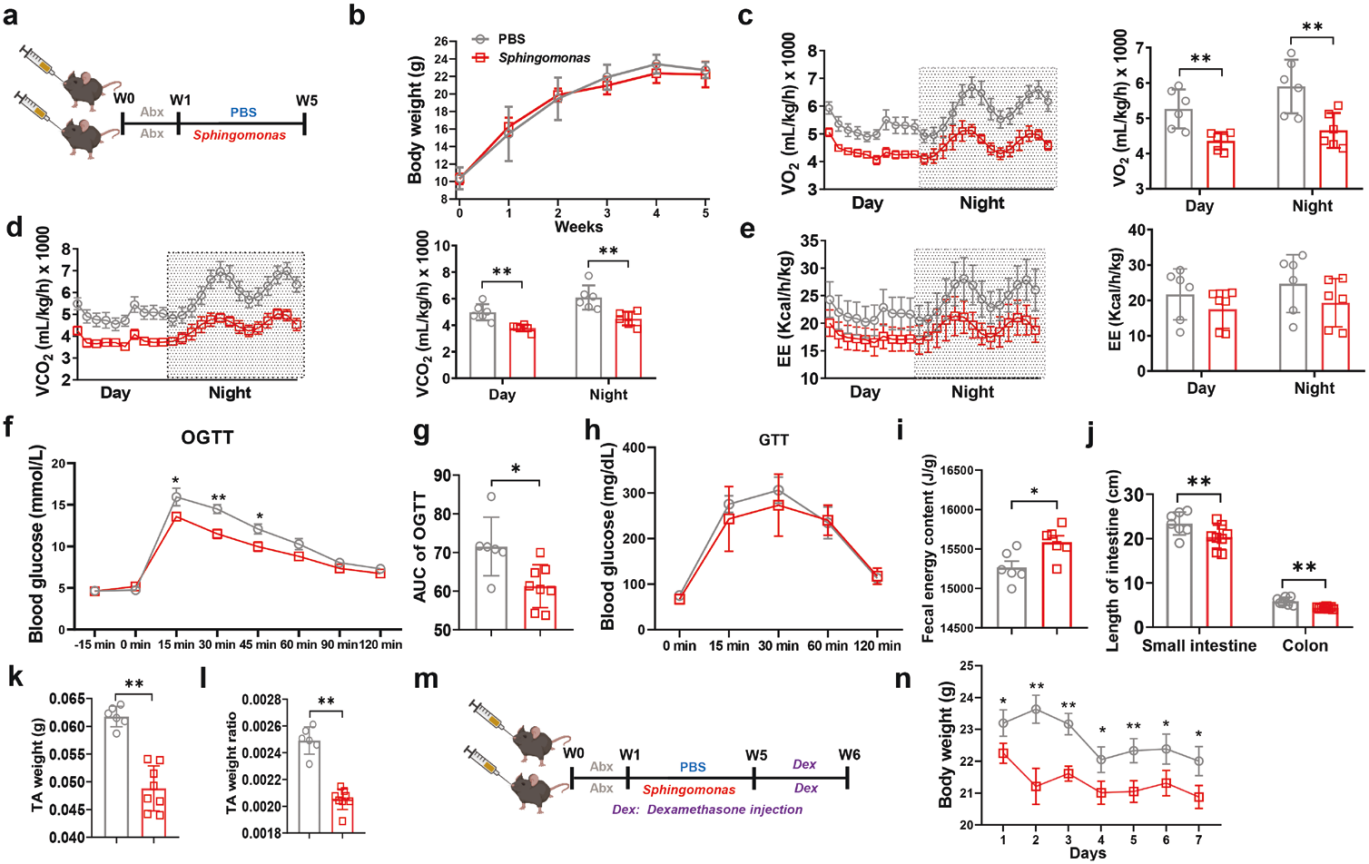
Effects of *Sphingomonas* on metabolism and weight loss under acute stress in the mice. (a) Diagram of *Sphingomonas* gavage experiment. Abx: antibiotic cocktail. (b) Cumulative BW of PBS and *Sphingomonas-*gavaged mice. (c–e) Oxygen volume (c), carbon dioxide volume (d), EE (e) of PBS and *Sphingomonas*-gavaged mice. (f–h) Glucose response curve of OGTT (f), AUC of OGTT (g), and glucose response curve of ipGTT for PBS and *Sphingomonas*-gavaged mice (h). (i) Fecal energy content of PBS and *Sphingomonas*-gavaged mice. (j) Small intestine and colon length of PBS and *Sphingomonas*-gavaged mice. (k and l) Weight (k) and ratio to BW (l) of TA muscle. (m and n) Diagram of dexamethasone treatment in PBS and *Sphingomonas*-gavaged mice (m) and BW changes (n). Dex: dexamethasone injection. The statistical significance is denoted as: ^*^P < 0.05, ^**^P < 0.01, ^***^P < 0.001.

To further explore the biological role of *S. paucimobilis* in metabolic regulation in different animal species, mice were daily gavaged with freshly prepared live *S. paucimobilis* (1 × 10^9^ live bacteria/day/mouse) and PBS for 5 weeks after the depletion of intestinal microbes using broad range antibiotics (Abx) administered in drinking water for 1 week (Fig. 6a). There was no significant difference in BW between the two groups (Fig. 6b), which was different from the results in piglets. Interestingly, the *S. paucimobilis*-colonized mice showed significant decreases in oxygen consumption rate at room temperature during both the light and dark phase, indicating that the gavage of *S. paucimobilis* led to metabolic dysregulation (Fig. 6c–e).

To further test the metabolic status of these mice, an oral glucose tolerance test (OGTT) was performed. As a result, *S. paucimobilis-*colonized mice showed a decrease in glucose peak after 15 min of glucose administration and the area under the curve was significantly lower than that of the PBS group (Fig. 6f and g). Interestingly, no difference was observed in the initial glucose peak under intraperitoneal administration of glucose (Fig. 6h), indicating that the *S. paucimobilis*-colonized mice might have weak absorption of glucose, which was consistent with the remarkable increase in fecal caloric content (Fig. 6i) and a significant decrease in the length of small intestine and colon (Fig. 6j) in *S. paucimobilis*-colonized mice. In addition, the *S. paucimobilis*-colonized mice had a pronounced decrease in the weight of tibialis anterior (TA) muscle (Fig. 6k and l). Thus, it could be hypothesized that the *S. paucimobilis*-colonized mice with metabolic disorder may be susceptible to weight loss. To test this hypothesis, glucocorticoid-induced mouse atrophy was used as an acute stress. As a result, a more significant weight loss was observed in the *Sphingomonas* group (Fig. 6m and n). These results suggested that the gavage of *S. paucimobilis* may reduce the length of the intestine to decrease glucose and energy absorption, leading to metabolic disorders and susceptibility to weight loss in mice, which might help to uncover the regulatory mechanism of *S. paucimobilis* on homeostasis and growth retardation.

### Adipose tissue is responsive to resident microbes in transcriptome and metabolome

**Figure 7:**
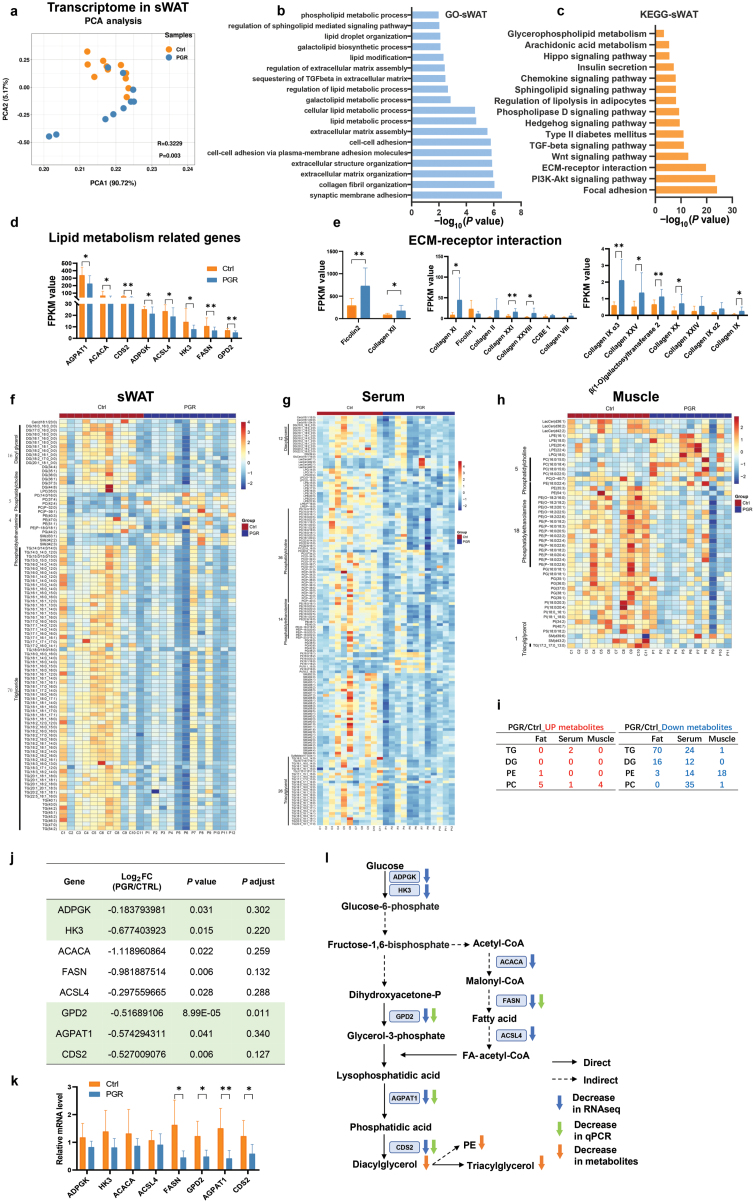
Transcriptome signatures of sWAT and lipidomics signatures of serum and sWAT in the Ctrl and PGR piglets. (a) Comparison of transcriptome in sWAT between the Ctrl and PGR piglets, revealed by PCA (Adonis *P* = 0.003). (b and c) GO analysis (b) and KEGG (c) in sWAT. (d and e) FPKM values of DEGs involved in lipid metabolism (d) and ECM-receptor interaction (e). (f–h) Lipidomics heatmap of sWAT (f), serum (g), and muscle (h). (i) Altered number of lipid types in sWAT, serum, and muscle. (j and k) Altered genes involved in TG and PE synthesis from RNA sequencing data of sWAT (j), and relative mRNA levels of DEGs (k). (l) Integration of DEGs and metabolites in TG and PE synthesis pathway. The statistical significance is denoted as: ^*^P < 0.05, ^**^P < 0.01, ^***^P < 0.001.

To understand how adipose tissue responds to the enrichment of specific bacterial taxa, RNA sequencing and lipidomics were performed. As shown in Fig. 7a, the transcriptome patterns of the Ctrl and PGR groups were significantly separated. As expected, genes enriched in lipid metabolism were significantly altered in the PGR group, which was consistent with the morphology of the adipose tissue (Fig. 7b–d). Interestingly, a number of genes clustered in the pathways of extracellular matrix (ECM) receptor interaction and focal adhesion were significantly upregulated in the sWAT of the PGR group (Fig. 7b, c, and e). Several intracellular metabolic pathways were also upregulated in the PGR group (Fig. 7b and c). Although the greatest transcriptional change did not occur in energy metabolism or lipid metabolism, the genes with the greatest fold changes were related to ECM receptor interaction, indicating a crosstalk between the adipose tissue and resident microbes [15] in the PGR group. However, no significant difference in muscle was found between the two groups, and lipid metabolism and ECM-related biological events were significantly changed as expected. The predominant pathways were related to skeletal system morphogenesis, microtubule-based protein transport, and transport along microtubule, suggesting that the main changes in protein synthesis and transportation occurred in the muscle (Supplementary Fig. S11a–c).

The adipose tissue has highly dynamic lipid metabolism and it is interesting to know whether the adipose tissue in the PGR group was responsive to resident microbes to affect lipid metabolism. To further dissect the response of adipose tissue and screen the differential metabolites, a lipidomics analysis was performed for the sWAT, serum, and muscle samples of the Ctrl and PGR groups (Supplementary Fig. S11d–f). Under growth retardation, a variety of lipid metabolites were significantly altered in the serum and sWAT (Fig. 7f and g). According to fat morphology in the PGR group shown in Fig. 1, the metabolites with significant decreases in sWAT and serum were related to triglycerides (TGs) and diacylglycerols (Fig. 7f and g), which are the main components of lipid droplets in adipose tissue. In the muscle, one kind of TGs decreased in the PGR group (Fig. 7h). Thus, the significant decrease in TGs may be a consequence of PGR.

To identify the functional lipids associated with growth performance, we analyzed the main lipids in sWAT, serum, and muscle. Except TG and diglyceride (DG), the main components of phospholipid, namely PE and phosphatidylcholine (PC), were found altered in PGR pigs. Consistent with TG and DG, a number of PE was shown to decrease in the three sources (Fig. 7i). Phospholipid on cellular membrane might be one of the main mediators for extracellular signals, which was in accordance with the activation of ECM receptor in the results of RNA sequencing (Fig. 7j and k). Therefore, we combined RNA sequencing and lipidomics (Fig. 7l). Key genes in the upstream of TG and PE synthesis decreased in the PGR group. Glycerol 3-phosphate dehydrogenase 2 (GPD2) converts dihydroxyacetone-P into glycerol-3-phosphate and cytidinediphosphate diacylglycerol synthase 2 (CDS2) converts phosphatidic acid into DG, which is the precursor for PE and TG. Consistently, some genes related to fatty acid (FA)-acetyl-CoA such as fatty acid synthase (*FASN*) also decreased, which provided a low level of substrate for lysophosphatidic acid. Together, upstream genes related to DG, TG, and PE decreased in the PGR group, indicating the low levels of these lipids in the PGR group in response to microbes’ residence. Thus, PE may act as a lipid target in regulating pig growth.

### The key metabolite phosphatidylethanolamine rescues growth retardation by suppressing the number of *Sphingomonas*

**Figure 8:**
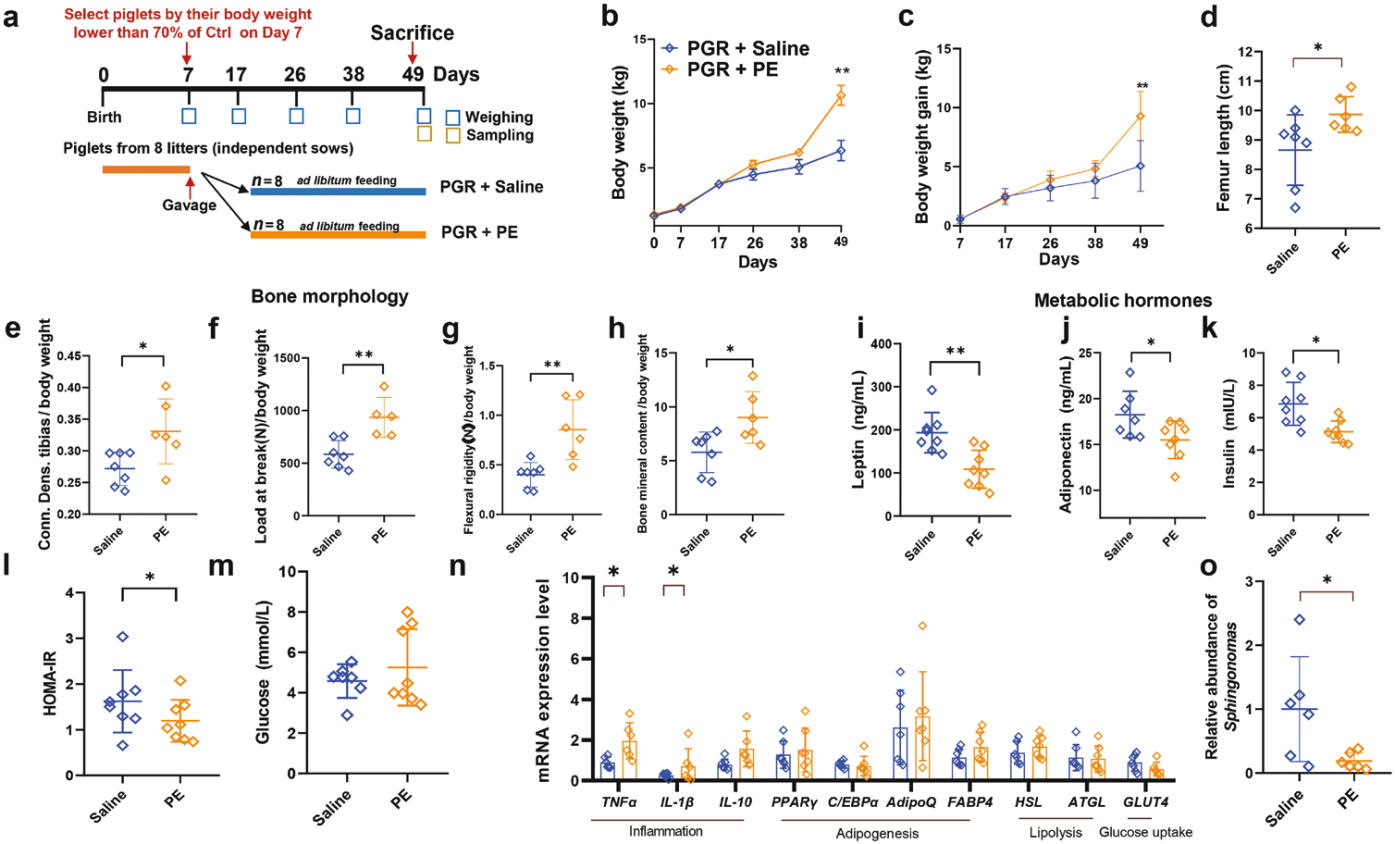
Effects of PE administration on growth performance and metabolic index of piglets. (a) Diagram of the experiment design. (b) BW of PGR + Saline and PGR + PE piglets. (c) BW gain of PGR + Saline and PGR + PE piglets. (d–h) Femur length (d), Conn. Dens. tibias (e), load at break (f), flexural rigidity (g), and bone mineral content (h) of PGR + Saline and PGR + PE piglets. (i–m) Levels of leptin (i), adiponectin (j), insulin (k), HOMA-IR (l), and glucose (m) in the serum of PGR + Saline and PGR + PE piglets. (n) mRNA levels of genes related to inflammation (*TNFα, IL-1β*, and *IL-10*), adipogenesis (*PPARγ*, *C/EBPα*, *AdipoQ*, and *FABP4*), lipolysis (*HSL* and *ATGL*), and glucose uptake (*GLUT4*) in sWAT assessed by qPCR. (o) Relative abundance of *Sphingomonas* detected by qPCR. The statistical significance is denoted as: ^*^P < 0.05, ^**^P < 0.01.

To further test the above hypothesis, we used PE + PGR model of piglets to clarify the role of PE in rescuing BW loss in the piglets (Fig. 8a). Intriguingly, PE could significantly improve BW (Fig. 8b–d) and bone development of the PGR piglets (Fig. 8e–h). To assess whether these effects could be attributed to the improvement of metabolic function in the adipose tissue, we determined the levels of adipokines and hormones and the expression of related genes. The administration of PE reversed the changes in adiponectin and leptin (Fig. 8i and j). Accordingly, PE administration decreased the levels of fasting insulin and the HOMA-IR index, indicating that PE may improve the insulin sensitivity of the PGR piglets (Fig. 8k–m). However, we only found a few differentially expressed genes (DEGs) for inflammation, but not for adipogenesis or glucose uptake (Fig. 8n). Next, to further elucidate whether PE is involved in decreasing the number of microbes, qPCR was carried out to detect *Sphingomonas*. As expected, the number of *Sphingomonas* decreased in sWAT in the PE-administrated group (Fig. 8o). These data suggested that the role of PE in regulating metabolism to promote pig growth may involve suppressing the number of *Sphingomonas* in sWAT.

## Discussion

Pigs have also been well recognized as an important biomedical model for studying human pediatric nutrition, including fetal, newborn, neonatal, and all other stages of early development [1], which offers several advantages in translational medical research. The role of the adipose tissue, a major endocrinal regulator of the whole body, in the development and metabolic regulation of PGR remains elusive [10]. Here, we employed the piglet PGR model to further demonstrate the function of the adipose tissue in PGR, suggesting a potential therapeutic target and strategies for growth retardation.

Our results revealed that PGR piglets had metabolic impairment with a smaller adipocyte size. In domestic pigs, the adipose tissue is taken as a vital index for meat quality, particularly the backfat, while few studies have been focused on the metabolic role of the adipose tissue [17]. Previously, we have demonstrated that the adipose tissue may act as an endocrinal regulator for the excellent reproductive performance of sows during pregnancy, indicating the importance of adipose tissue in the metabolism of pigs [37]. During growth and development, the metabolic function of adipose tissue has been largely ignored. Our results demonstrated that the key adipokines excreted by the adipose tissue under PGR showed a similar trend to those excreted under starvation [10]. Adiponectin, the most extensively studied adipokine, showed a significant increase in PGR and *Sphingomonas*-induced PGR models, while a remarkable decrease in the PE + PGR group. The role of adiponectin has been well documented in the metabolism of adults but not in young animals yet. In some weight loss models, the level of adiponectin increased in children [38]. Notably, adiponectin has been identified as a positive regulator of insulin in animal models and humans [6]. Considering the low glucose uptake and insulin resistance of the PGR piglets, adiponectin may decrease the level of circulating glucose, which may help to increase insulin sensitivity. These results together with previous findings in children and pigs demonstrate the metabolic role of adipose tissue in the PGR model.

Some studies have reported the presence of bacteria in adipose tissue in chronic inflammation, obesity, and diabetes [12, 13]. Recently, it was reported that *Proteobacteria* and *Firmicutes* are the predominant bacterial phyla in the adipose tissue [14]. Similar results were also obtained in pig adipose tissue, and the top three phyla were *Proteobacteria*, *Firmicutes*, and *Bacteroidetes* in both the adipose tissue and blood. In the four tissues analyzed by 16S rRNA gene sequencing, *Proteobacteria* was a common intra-tissue bacterium, which is similar to the findings in previous studies [12, 14]. The predominance of *Proteobacteria* indicated its specific function in the adipose tissue. Thus, we further focused on the class and genera in *Proteobacteria* to find the microbe with top relative abundance, namely *Sphingomonas*, which was then confirmed by CARD-FISH and qPCR. To discover the key microbes related to metabolism in the adipose tissue, we had attempted but failed to isolate and culture *Sphingomonas* from pig and mouse adipose tissue, due to the small number of the bacteria and the high abundance of fat in this special tissue. A previous study has reported the role of one defined species of *Sphingomonas*, *S. paucimobilis*, in inflammatory bowel disease [35], suggesting the functional link between *S. paucimobilis* and gut health. Further, *S. paucimobilis* isolated by other groups [39] was used here for establishing bacteria-induced PGR model, which might be a limitation of this study. Surprisingly, *Sphingomonas*-induced PGR showed high similarity to the PGR model including bone morphology, adipose morphology, and metabolic index in the serum, indicating the role of *S. paucimobilis* in restricting BW gain. These data suggest the presence of microbes in adipose tissue and their metabolic function in regulating piglet growth.

Next, we attempted to understand the response of adipose tissue to microbes in PGR piglets. RNA sequencing revealed that the ECM-related genes were the most highly expressed in transcriptome, implying a crosstalk between the adipose tissue and microbes, which has also been reported by other studies [15]. As a primary organ for energy storage in the body, the adipose tissue undergoes lipolysis and lipogenesis for a balance all the time [31]. The metabolism of lipids may function as a signal from the adipose tissue to regulate whole-body homeostasis [6]. PE is a positive regulator of humoral immunity [40], cell proliferation [41], and insulin sensitivity [42, 43], which is still largely unknown to us. PE administration could improve the metabolic function of adipose tissue at least in terms of adiponectin level and HOMA-IR index. Besides, microbiology analysis in the adipose tissue revealed that PE administration decreased the abundance of *Sphingomonas* in the PGR group as detected by qPCR. These results suggest that variations in the key metabolites in the adipose tissue are the result of host-microbe crosstalk under PGR, indicating that “metabolite-bacteria-organ development-whole body” may be a novel route to ameliorate pig growth retardation as well as human growth restriction. However, which PE would be the valuable molecule for precision medicine in growth promoting will be the next complex questions to answer. Although commercial availability is an immense obstacle for further exploring, the functional phospholipids may provide a novel avenue for the treatment of metabolic diseases.

**Figure 9:**
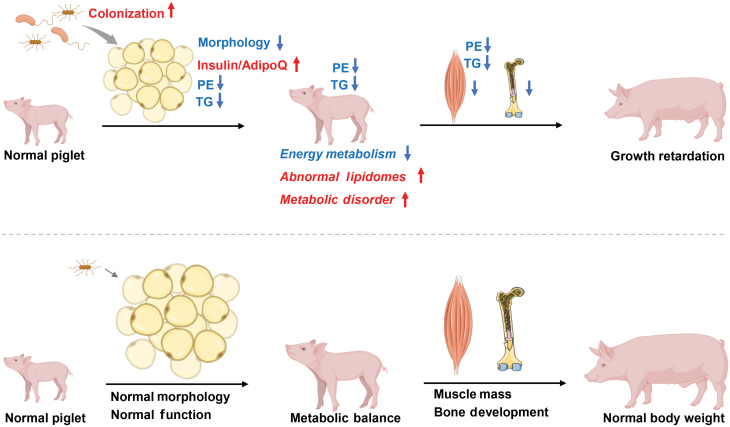
Schematic illustration of PGR-induced *S. paucimobilis* resided in the adipose tissue. *S. paucimobilis* residing in the adipose tissue decreases the morphology of the adipose tissue, leading to abnormal lipidomes and metabolic disorders and exacerbating pig growth retardation.

Overall, the present study highlights the relationship between the adipose tissue-derived microbe and piglet growth, and provides the therapeutic target and potential way to rescue PGR (Fig. 9). However, there are still some limitations here. An interesting and challenging question is to clarify where *Sphingomonas* is derived from. Both intestine and lung are connected to the exterior of the body [16], which may be two distinct ways for microbe translocation. The specific molecular mechanisms of microbe-induced imbalance of metabolism and the PE-induced increase in BW remain elusive, which need to be elucidated by further experiments.

## Materials and methods

### Study design

For piglet trial 1, crossbred piglets (Landrace × Yorkshire), which are widely used in commercial pig production, were raised in this study. As a result, 30 litters of newborn piglets were generated from 30 sows, with a total of 370 newborns (average 12 newborns per litter) of the same genetic background. All piglets were fed with sow milk during a suckling period (before Day 26) and weaned on Day 26. Then, they were separated from sows, and fed with a nursery diet during the post-weaning period. During the post-weaning period, all piglets were housed together. Water and feed were provided *ad libitum*. All piglets were raised together and received enough feed, which were adequately supported, and would not compete for feed during any growth stage.

BW of these piglets was recorded on Day 1, 7, 14, and 21 before weaning, and Day 28, 35, 42, 49, and 60 after weaning. All diets were antibiotics-free, and the dietary nutrients met the NRC (2012) recommendation. Water was provided *ad libitum* from nipple drinkers. On Day 60, 30 Ctrl piglets and 30 PGR piglets were sacrificed for sampling. sWAT, aWAT, whole blood, and liver were taken under sterile conditions and assessed using different omics datasets, including 16S rRNA gene sequencing, RNA sequencing, and metabolomes (lipidomics). Preoperative preparation of the piglets and surgical facility was performed as described previously [44] using a standardized procedure by a team of trained technicians. Samples were collected as previously described [45] by pathology-trained personnel wearing surgical masks and sterile gloves and using sterile disposable surgical instruments. Neck vein blood was sampled using a sterile needle and syringe and stored at –80°C. The piglets were removed to a chemical and ultraviolet sterilized, class II B2 biosafety cabinet and placed into an autoclaved and blaze-disinfected salver. The piglets were then euthanized, and the subcutaneous and abdominal fat were collected with sterile scalpel and forceps.

For piglet trial 2 (bacterial transplantation), newborn piglets from 8 litters were recorded to validate the discovery from trial 1. Piglets were divided into two groups with oral administration every day from the ages of 7 to 49 days (*n* = 8 in each group): healthy piglets received normal saline (Saline group); healthy piglets gavaged with 1 × 10^9^ live bacteria per day per piglet for 6 weeks from Day 7 after birth to Day 49 (*Sphingomonas* group). On Day 49, the piglets were sacrificed for sampling.

For piglet trial 3 (validation), newborn piglets from 8 litters in trial 2 were recorded to validate the discovery from trial 1. Piglets were divided into four groups with oral administration every day from Day 7 to Day 49 (*n* = 8 in each group): healthy piglets received normal saline (Ctrl + Saline); healthy piglets received PE (Ctrl + PE); PGR piglets received normal saline (PGR + Saline); PGR piglets received PE (PGR + PE). Piglets were orally administrated every day with 0.78 g PE dissolved in normal saline during suckling period and 2.11 g PE during post-weaning period. On Day 49, piglets were sacrificed for sampling. The dosage of PE was calculated by the supplementation of PC [46].

For both trials 2 and 3, the BW of piglets was recorded on Day 7, 17, 26, 38, and 49. Piglets were sacrificed on Day 49 for sampling. The relative weight of each organ was calculated as the organ weight divided by BW (g/kg). Blood samples (serum) were collected from a jugular vein and stored at –80°C for further processing. The sWAT samples were fixed in 4% paraformaldehyde. Femur bones were collected for determination of bone density and bone strength with dual-energy x-ray absorptiometry.

For transplantation in mice, all specific pathogen free male C57BL/6J mice were fed in 12 h light/12 h night cycle in the animal center of Huazhong Agricultural University (Wuhan, China), and provided with a standard laboratory diet and clean water. Each group of mice was kept in separate cages. All mice were allocated to experimental groups based on their BW to ensure equal starting points. Bacterial transplantation experiment was conducted on 3-week-old weaned mice by oral gavage with *S. paucimobilis* (10^9^ CFU/day). Before transplantation, antibiotic treatment was employed for mice as follows. Mice were treated with antibiotics (1 g/L metronidazole, 1 g/L neomycin, 1 g/L ampicillin, and 0.5 g/L vancomycin) dissolved in the drinking water for 1 week. *S. paucimobilis* (ATCC 29837) [39] was purchased from China General Microbiological Culture Collection Center (Beijing, China). Dexamethasone gavage, which contributes to the onset of muscle atrophy to inhibit growth rate [47], was performed to see the effect of *S. paucimobilis* in susceptibility to acute stress in mice.

### 16S rRNA gene sequencing analysis

The tissue microbes were analyzed as previously described [15]. Briefly, total bacterial DNA was extracted using the DNA stool mini kit (Tiangen, Beijing, China). The V3–V4 region of bacterial 16S rRNA gene was amplified and sequenced by Shanghai Personal Biotechnology Limited Company (Shanghai, China) using an Illumina MiSeq (Illumina, USA) sequencing platform. The sequencing reads were analyzed by QIIME2 (quantitative insights into microbial ecology, via QIIME2 website) analysis pipeline as previously described [48]. In brief, paired-end reads were joined, demultiplexed, and quality controlled with DADA2 plugin, and then the ASV table was obtained [49]. The taxonomic assignment of ASV table was performed with the q2-feature-classifier, which was trained for the used primers using the 99% OTU data set of the SILVA Release 138 [50]. All samples were then rarefied for subsequent diversity analysis.

α-diversity was calculated with R package “vegan” [51]. β-diversity was calculated with PCA (principle component analysis) performed with function princomp in R, and permutational multivariate analysis of variance (PERMANOVA) was carried out with ANOSIM (analysis of similarity) [52]. Microbial enrichment analysis was performed with linear discriminant analysis (LDA) effect size (LEfSe) with the LDA threshold of 3 [53].

### RNA sequencing analysis

RNA extraction and RNA sequencing analysis were performed by Majorbio Bio-pharm Technology as previously described [54]. Total RNA of sWAT was extracted using the total RNA extractor (Trizol) kit (B511311, Sangon, China) according to the manufacturer’s protocol, and treated with RNase-free DNase I to remove genomic DNA contamination. A total of 2 μg RNA per sample was used as input for RNA sequencing library construction. Sequencing libraries were generated using VAHTSTM mRNA-seq V2 Library Prep Kit for Illumina®, following the manufacturer’s recommendations, and index codes were added to attribute the sequences to each sample. The libraries were then quantified and pooled. Paired-end sequencing of the library was performed on the HiSeq XTen sequencers (Illumina, San Diego, CA). FastQC (version 0.11.2) [55] was used for evaluating the quality of sequenced data. Raw reads were filtered by Trimmomatic (version 0.36) [56]. Clean reads were mapped to the reference genome by HISAT2 (version 2.0) [57] with default parameters. RSeQC (version 2.6.1) [58] was used to run statistics on the alignment results. Gene expression values of the transcripts were computed by StringTie (version 1.3.3b) with parameters: -e [59]. RSEM (via the website of "RSEM (RNA-Seq by Expectation-Maximization)") [60] was used to quantify gene abundance. DESeq2 (version 1.12.4) [61] was used to determine DEGs. Genes were considered as significantly differentially expressed when *P* value < 0.05. In addition, function-enrichment analysis including gene ontology (GO) and KEGG was performed to identify DEGs significantly enriched in GO terms and metabolic pathways at *P* value < 0.05 compared with the whole-transcriptome background. GO functional enrichment and KEGG pathway analysis were carried out by Goatools and KOBAS [62]. The data were analyzed on the free online platform of Majorbio Cloud Platform.

### Lipidomics assay

Lipid extraction and mass spectrometry-based lipid detection were performed by Applied Protein Technology. A separate sample was taken from each group and mixed equally to create a pooled QC sample. QC samples were inserted into the analysis queue to evaluate the system stability and data reliability during the whole experimental process. LC–MS/MS analysis was performed on a Q Exactive plus mass spectrometer (Thermo Scientific) coupled to a UHPLC Nexera LC-30A (SHIMADZU). Full-scan spectra were collected in mass-to-charge ratio (m/z) ranges of 200–1800 and 250–1800 for positive and negative ion modes, respectively. The mass-to-charge ratio of lipid molecules to lipid fragments was collected with the following method: after each full scan, 10 fragment patterns (MS2 scan, HCD) were collected. Lipid identification (secondary identification), peak extraction, peak alignment, and quantification were performed with LipidSearch software (version 4.1, Thermo Scientific™). In the extracted ion features, only the variables with more than 50% of the nonzero measurement values in at least one group were kept.

### qPCR

qPCR was carried out as previously described [29]. For gene expression analysis, total RNA was extracted from the adipose tissue using Trizol reagent (Thermo Fisher Scientific), and reverse transcribed using random primers and M-MLV reverse-transcriptase (Thermo Fisher Scientific). For *S. Paucimobilis* analysis, total DNA was purified from sWAT. β-actin and 18S were used as the internal references for qPCR respectively. SYBR premix EX Taq (Takara) was used for qPCR analysis, which was performed on a Roche 480 real-time PCR system (Roche). The primers used for qPCR analysis are listed in Supplementary Tables S1 and S2.

### CARD-FISH

Visualization of bacterial cells in the tissues was carried out using CARD-FISH as previously described [12, 35, 63]. Briefly, the tissues of mice and pigs were fixed in 4% paraformaldehyde, and then embedded in paraffin and sectioned. De-paraffinized sections were sequentially treated with permeability mixture buffer (Proteinase K buffer, SDS buffer, lysozyme buffer, and achromopeptidase buffer) for 1 h at 37°C to achieve permeabilization. The slides were incubated in hybridization buffer with HRP-labeled CARD-FISH probes (0.17 ng/mL) for 3 h at 37°C in a humidified chamber and then washed gently for three times in wash buffer and 1 × PBS for 15 min at room temperature. CARD-FISH was performed by incubating the sections for 20 min at 37°C in an amplification buffer. The samples were then washed for three times with 1 × PBS and stained with DAPI (1 µg/mL) for 10 min at room temperature. Images were acquired on a Leica inverted fluorescence microscope. The probes used for CARD-FISH are listed in Supplementary Table S2. The top 100 of the relatively abundant microbes are listed in Supplementary Tables S3–S6.

### Measurement of serum hormone levels

The porcine plasma levels of leptin, adiponectin, insulin, and glucose were determined using commercial ELISA kit according to the manufacturer’s protocol (Jiangsu Meimian industrial Co., Ltd., Jiangsu, China).

### Bacteria culture and identification

In brief, for the isolation of bacteria, pig adipose tissue pieces (~0.05 g) were homogenized with glass homogenizer in 1 mL cold PBS under sterile conditions. PBS was treated with the same process to evaluate contamination from environment. Homogenized tissue samples were filtered by 70 μm cell strainer (Biosharp, BS-70-CS), and 100 μL of the samples were subsequently plated on Columbia blood agar base plate (Columbia blood agar (OXOID, CM0331B) + 5% sheep blood (Solarbio, TX0030)) at 37°C [34]. For further identification of bacteria, colonies were picked and streaked to get single colonies. Next, the single colony was picked into the liquid medium with 20 μL and run PCR using specific primers for *Sphingomonas* and *S. paucimobilis* (listed in Supplementary Table S2) [36]. The PCR product was identified by gel electrophoresis and sequencing [34].

### Whole genome sequence analysis of bacteria

Single colonies were picked from streaked plates and inoculated into LB broth. After overnight culture, bacterial DNA was extracted using a soil DNA Kit (OMEGA, D5625) according to the manufacturer’s instructions. Sequencing libraries were prepared using the NEBNext® Ultra™ DNA Library Prep Kit for Illumina (NEB, USA) following the manufacturer’s recommendations, and index codes were added to attribute sequences to each sample. The quality of libraries was analyzed using the Agilent Bioanalyzer before pooling. *S. paucimobilis* isolates derived from the PGR piglets were sequenced on the NovaSeq 6000 sequencing using a 2 × 150 bp v2 kit (Illumina).

### Oral glucose tolerance test and intraperitoneal glucose tolerance test

Mice were fasted overnight for about 16 h and orally loaded with glucose (2.5 g/kg BW). The blood was collected from the tail vein at –15, 0, +15, +30, +45, +60, +90, and +120 min to measure plasma glucose levels [64]. For intraperitoneal glucose tolerance test (ipGTT), each mouse was weighed and intraperitoneally injected with glucose at 2.5 g/kg BW [65].

### Metabolic cage analysis

All mice had a 3-day adaption to single caging. Air-tight cages were designed for metabolic phenotyping in an open-circuit indirect calorimetric system. The sampling interval for each cage was 2 min, with repetition every 18 min. A total of 72 data points for food intake, O_2_ consumption, and CO_2_ production were measured using a 2-dimensional infrared light-bean. Energy expenditure (EE) and oxygen consumption (V_O2_) were calculated using the manufacture’s software and values were corrected for body mass [66].

### Bomb calorimetry

Feces were all collected for 24 h, and dried to constant weight at 60°C. Fecal energy content of every mouse was measured by a bomb calorimeter (IKA C200, Staufen, Germany) [66].

### Histology

Adipose tissue samples were fixed immediately in 4% paraformaldehyde. Paraffin-embedded adipose tissues were sectioned into 6-µm slides and stained with H&E.

### Statistical analysis

Data were presented as means ± SD. Paired and unpaired two-tailed Student’s *t*-test and two-way ANOVA were used to calculate statistical significance. *P* < 0.05 was considered as statistically significant. Outliers were tested by the Grubbs outlier test and excluded below a threshold of *P* = 0.001 [67]. One outlier of serum insulin level in the PGR group (Grubbs G = 2.75) was excluded, and the result of Student’s *t*-test changed from 0.060 to 0.0512 in Fig. 1. Similarly, one outlier value of AdipoQ mRNA expression level in *Sphingomonas* group was removed (Grubbs *G* = 1.733). The result of Student’s *t*-test changed from 0.049 to 0.006 in Fig. 5. An outlier in the serum glucose level of PGR + Saline group was excluded (Grubbs *G* = 2.32) in Fig. 8. The result of Student’s *t*-test changed from 0.951 to 0.399. Statistical calculations were performed using GraphPad Prism 8.

## Supplementary Material

load052_suppl_Supplementary_Figures_S1-S11_Tables_S1-S6

## Data Availability

All data are made available in the main text or the Supplementary materials in the repositories upon publication. The accession number for 16S rRNA sequence data reported in this paper is NCBI BioProject: PRJNA823816 and PRJNA823821. The accession number for RNA sequence data reported in this paper is NCBI BioProject: PRJNA824965, PRJNA824968, and PRJNA824969. The lipidomics data have been deposited into the CNGB Sequence Archive (CNSA) of China National GeneBank DataBase (CNGBdb) with accession number CNP0002883.

## References

[1] Lunney JK, Van Goor A, Walker KE et al. Importance of the pig as a human biomedical model. Sci Transl Med 2021;13:eabd5758. 34818055 10.1126/scitranslmed.abd5758

[2] Wu G, Fanzo J, Miller DD et al. Production and supply of high‐quality food protein for human consumption: sustainability, challenges, and innovations. Ann N Y Acad Sci 2014;1321:1–19. 25123207 10.1111/nyas.12500

[3] Patience JF, Rossoni-Serão MC, Gutiérrez NA. A review of feed efficiency in swine: biology and application. J Anim Sci Biotechnol 2015;6:33. 26251721 10.1186/s40104-015-0031-2 PMC4527244

[4] Burrin D, Sangild PT, Stoll B et al. Translational advances in pediatric nutrition and gastroenterology: new insights from pig models. Annu Rev Anim Biosci 2020;8:321–54. 32069436 10.1146/annurev-animal-020518-115142

[5] Chouchani ET, Kajimura S. Metabolic adaptation and maladaptation in adipose tissue. Nat Metab 2019;1:189–200. 31903450 10.1038/s42255-018-0021-8 PMC6941795

[6] Morigny P, Boucher J, Arner P et al. Lipid and glucose metabolism in white adipocytes: pathways, dysfunction and therapeutics. Nat Rev Endocrinol 2021;17:276–95. 33627836 10.1038/s41574-021-00471-8

[7] Rosen ED, Spiegelman BM. What we talk about when we talk about fat. Cell 2014;156:20–44. 24439368 10.1016/j.cell.2013.12.012 PMC3934003

[8] Kuzawa CW. Adipose tissue in human infancy and childhood: an evolutionary perspective. Am J Phys Anthropol 1998;107:177–209. 10.1002/(sici)1096-8644(1998)107:27+<177::aid-ajpa7>3.0.co;2-b 9881526

[9] Kotnik P, Posovszky PF, Wabitsch M. Endocrine and metabolic effects of adipose tissue in children and adolescents. SVN J Public Health 2015;54:131. 10.1515/sjph-2015-0020 PMC4820166 27646920

[10] Wensveen FM, Valentić S, Šestan M et al. Interactions between adipose tissue and the immune system in health and malnutrition. Semin Immunol 2015;27:322–33. 26603491 10.1016/j.smim.2015.10.006

[11] Amar J, Chabo C, Waget A et al. Intestinal mucosal adherence and translocation of commensal bacteria at the early onset of type 2 diabetes: molecular mechanisms and probiotic treatment. EMBO Mol Med 2011;3:559–72. 21735552 10.1002/emmm.201100159 PMC3265717

[12] Massier L, Chakaroun R, Tabei S et al. Adipose tissue derived bacteria are associated with inflammation in obesity and type 2 diabetes. Gut 2020;69:1796–806. 32317332 10.1136/gutjnl-2019-320118

[13] Anhê FF, Jensen BAH, Varin TV et al. Type 2 diabetes influences bacterial tissue compartmentalisation in human obesity. Nat Metab 2020;2:233–42. 32694777 10.1038/s42255-020-0178-9

[14] He Z, Wu J, Gong J et al. Microbiota in mesenteric adipose tissue from Crohn’s disease promote colitis in mice. Microbiome 2021;9:228. 34814945 10.1186/s40168-021-01178-8 PMC8609859

[15] Ha CWY, Martin A, Sepich-Poore GD et al. Translocation of viable gut microbiota to mesenteric adipose drives formation of creeping fat in humans. Cell 2020;183:666–83.e17. 32991841 10.1016/j.cell.2020.09.009 PMC7521382

[16] McPherson AC, Pandey SP, Bender MJ et al. Systemic immunoregulatory consequences of gut commensal translocation. Trends Immunol 2021;42:137–50. 33422410 10.1016/j.it.2020.12.005 PMC10110348

[17] Miller E, Ullrey D. The pig as a model for human nutrition. Annu Rev Nutr 1987;7:361–82. 3300739 10.1146/annurev.nu.07.070187.002045

[18] Roura E, Koopmans SJ, Lallès JP et al. Critical review evaluating the pig as a model for human nutritional physiology. Nutr Res Rev 2016;29:60–90. 27176552 10.1017/S0954422416000020

[19] Stoltz DA, Meyerholz DK, Pezzulo AA et al. Cystic fibrosis pigs develop lung disease and exhibit defective bacterial eradication at birth. Sci Transl Med 2010;2:29ra31. 10.1126/scitranslmed.3000928 PMC2889616 20427821

[20] Virdi V, Coddens A, De Buck S et al. Orally fed seeds producing designer IgAs protect weaned piglets against enterotoxigenic *Escherichia coli* infection. Proc Natl Acad Sci USA 2013;110:11809–14. 23801763 10.1073/pnas.1301975110 PMC3718133

[21] Ferenc K, Pietrzak P, Godlewski MM et al. Intrauterine growth retarded piglet as a model for humans–studies on the perinatal development of the gut structure and function. Reprod Biol 2014;14:51–60. 24607255 10.1016/j.repbio.2014.01.005

[22] Smith MI, Yatsunenko T, Manary MJ et al. Gut microbiomes of Malawian twin pairs discordant for kwashiorkor. Science 2013;339:548–54. 23363771 10.1126/science.1229000 PMC3667500

[23] Forgie AJ, Drall KM, Bourque SL et al. The impact of maternal and early life malnutrition on health: a diet-microbe perspective. BMC Med 2020;18:135. 32393275 10.1186/s12916-020-01584-z PMC7216331

[24] Charbonneau MR, O’Donnell D, Blanton LV et al. Sialylated milk oligosaccharides promote microbiota-dependent growth in models of infant undernutrition. Cell 2016;164:859–71. 26898329 10.1016/j.cell.2016.01.024 PMC4793393

[25] Chang HW, McNulty NP, Hibberd MC et al. Gut microbiome contributions to altered metabolism in a pig model of undernutrition. Proc Natl Acad Sci USA 2021;118:e2024446118. 34001614 10.1073/pnas.2024446118 PMC8166152

[26] Spurlock ME, Gabler NK. The development of porcine models of obesity and the metabolic syndrome. J Nutr 2008;138:397–402. 18203910 10.1093/jn/138.2.397

[27] Renner S, Blutke A, Clauss S et al. Porcine models for studying complications and organ crosstalk in diabetes mellitus. Cell Tissue Res 2020;380:341–78. 31932949 10.1007/s00441-019-03158-9

[28] Qi M, Tan B, Wang J et al. The microbiota–gut–brain axis: a novel nutritional therapeutic target for growth retardation. Crit Rev Food Sci Nutr 2022;62:4687–92. 10.1080/10408398.2021.1879004 33523720

[29] Qi M, Wang J, Tan B et al. Postnatal growth retardation is associated with intestinal mucosa mitochondrial dysfunction and aberrant energy status in piglets. J Cell Mol Med 2020;24:10100–11. 32667125 10.1111/jcmm.15621 PMC7520312

[30] Knauer MT, Hostetler CE. US swine industry productivity analysis, 2005 to 2010. J Swine Health Prod 2013;21:248–52.

[31] Ghaben AL, Scherer PE. Adipogenesis and metabolic health. Nat Rev Mol Cell Biol 2019;20:242–58. 30610207 10.1038/s41580-018-0093-z

[32] Moreno‐Reyes R, Egrise D, Nève J et al. Selenium deficiency‐induced growth retardation is associated with an impaired bone metabolism and osteopenia. J Bone Miner Res 2001;16:1556–63. 11499879 10.1359/jbmr.2001.16.8.1556

[33] Katsuki A, Sumida Y, Gabazza EC et al. Homeostasis model assessment is a reliable indicator of insulin resistance during follow-up of patients with type 2 diabetes. Diabetes Care 2001;24:362–5. 11213893 10.2337/diacare.24.2.362

[34] Fu A, Yao B, Dong T et al. Tumor-resident intracellular microbiota promotes metastatic colonization in breast cancer. Cell 2022;185:1356–72.e26. 35395179 10.1016/j.cell.2022.02.027

[35] Sun D, Bai R, Zhou W et al. Angiogenin maintains gut microbe homeostasis by balancing α-Proteobacteria and Lachnospiraceae. Gut 2021;70:666–76. 32843357 10.1136/gutjnl-2019-320135 PMC7904960

[36] Leung K, Chang Y, Gan Y et al. Detection of *Sphingomonas* spp in soil by PCR and sphingolipid biomarker analysis. J Ind Microbiol Biotechnol 1999;23:252–60. 11423941 10.1038/sj.jim.2900677

[37] Song T, Lu J, Deng Z et al. Maternal obesity aggravates the abnormality of porcine placenta by increasing N^6^-methyladenosine. Int J Obes 2018;42:1812–20. 10.1038/s41366-018-0113-2 29795472

[38] Jeffery AN, Murphy MJ, Metcalf BS et al. Adiponectin in childhood. Int J Pediatr Obes 2008;3:130–40. 19086185 10.1080/17477160801954538

[39] Yabuuchi E, Yano I, Oyaizu H et al. Proposals of *Sphingomonas paucimobilis* gen. nov. and comb. nov., *Sphingomonas parapaucimobilis* sp. nov., *Sphingomonas yanoikuyae* sp. nov., *Sphingomonas adhaesiva* sp. nov., *Sphingomonas capsulata* comb., nov., and two genospecies of the genus *Sphingomonas*. Microbiol Immunol 1990;34:99–119. 2111872 10.1111/j.1348-0421.1990.tb00996.x

[40] Fu G, Guy CS, Chapman NM et al. Metabolic control of T_FH_ cells and humoral immunity by phosphatidylethanolamine. Nature 2021;595:724–9. 34234346 10.1038/s41586-021-03692-z PMC8448202

[41] Che H, Zhang L, Ding L et al. EPA-enriched ethanolamine plasmalogen and EPA-enriched phosphatidylethanolamine enhance BDNF/TrkB/CREB signaling and inhibit neuronal apoptosis *in vitro* and *in vivo*. Food Funct 2020;11:1729–39. 32043504 10.1039/c9fo02323b

[42] van der Veen JN, Lingrell S, McCloskey N et al. A role for phosphatidylcholine and phosphatidylethanolamine in hepatic insulin signaling. FASEB J 2019;33:5045–57. 30615497 10.1096/fj.201802117R

[43] Lee S, Norheim F, Gulseth HL et al. Skeletal muscle phosphatidylcholine and phosphatidylethanolamine respond to exercise and influence insulin sensitivity in men. Sci Rep 2018;8:7885. 29760520 10.1038/s41598-018-26061-9 PMC5951798

[44] Riou Y, Gouet P, Dubourguier HC et al. Techniques for obtaining, fistulization and rearing of axenic or gnotoxenic lambs, kids and calves. Ann Rech Vet 1977;8:13–24. 879673

[45] Seferovic MD, Pace RM, Carroll M et al. Visualization of microbes by 16S in situ hybridization in term and preterm placentas without intraamniotic infection. Am J Obstet Gynecol 2019;221:146.e1–146.e23. 10.1016/j.ajog.2019.04.036 PMC10357491 31055031

[46] Ross RG, Hunter SK, Hoffman MC et al. Perinatal phosphatidylcholine supplementation and early childhood behavior problems: evidence for CHRNA7 moderation. Am J Psychiatry 2016;173:509–16. 26651393 10.1176/appi.ajp.2015.15091188 PMC5892450

[47] Sakai H, Kimura M, Tsukimura Y et al. Dexamethasone exacerbates cisplatin‐induced muscle atrophy. Clin Exp Pharmacol Physiol 2019;46:19–28. 30137654 10.1111/1440-1681.13024

[48] Caporaso JG, Kuczynski J, Stombaugh J et al. QIIME allows analysis of high-throughput community sequencing data. Nat Methods 2010;7:335–6. 20383131 10.1038/nmeth.f.303 PMC3156573

[49] Callahan BJ, McMurdie PJ, Rosen MJ et al. DADA2: high-resolution sample inference from Illumina amplicon data. Nat Methods 2016;13:581–3. 27214047 10.1038/nmeth.3869 PMC4927377

[50] Quast C, Pruesse E, Yilmaz P et al. The SILVA ribosomal RNA gene database project: improved data processing and web-based tools. Nucleic Acids Res 2012;41:D590–6. 23193283 10.1093/nar/gks1219 PMC3531112

[51] Dixon P. VEGAN, a package of R functions for community ecology. J Veg Sci 2003;14:927–30.

[52] McArdle BH, Anderson MJ. Fitting multivariate models to community data: a comment on distance‐based redundancy analysis. Ecology 2001;82:290–7.

[53] Segata N, Izard J, Waldron L et al. Metagenomic biomarker discovery and explanation. Genome Biol 2011;12:R60. 21702898 10.1186/gb-2011-12-6-r60 PMC3218848

[54] Xu Z, You W, Zhou Y et al. Cold-induced lipid dynamics and transcriptional programs in white adipose tissue. BMC Biol 2019;17:74. 31530289 10.1186/s12915-019-0693-x PMC6749700

[55] Andrews S. FastQC: A Quality Control Tool for High Throughput Sequence Data: Babraham Bioinformatics. Cambridge: Babraham Institute, 2010.

[56] Bolger AM, Lohse M, Usadel BT. A flexible trimmer for Illumina sequence data. Bioinformatics 2014;30:2114–20. 24695404 10.1093/bioinformatics/btu170 PMC4103590

[57] Kim D, Langmead B, Salzberg SL. HISAT: a fast spliced aligner with low memory requirements. Nat Methods 2015;12:357–60. 25751142 10.1038/nmeth.3317 PMC4655817

[58] Wang L, Wang S, Li W. RSeQC: quality control of RNA-seq experiments. Bioinformatics 2012;28:2184–85. 22743226 10.1093/bioinformatics/bts356

[59] Pertea M, Pertea GM, Antonescu CM et al. StringTie enables improved reconstruction of a transcriptome from RNA-seq reads. Nat Biotechnol 2015;33:290–5. 25690850 10.1038/nbt.3122 PMC4643835

[60] Li B, Dewey CN. RSEM: accurate transcript quantification from RNA-seq data with or without a reference genome. BMC Bioinformatics 2011;12:323. 21816040 10.1186/1471-2105-12-323 PMC3163565

[61] Love MI, Huber W, Anders S. Moderated estimation of fold change and dispersion for RNA-seq data with DESeq2. Genome Biol 2014;15:550. 25516281 10.1186/s13059-014-0550-8 PMC4302049

[62] Xie C, Mao X, Huang J et al. KOBAS 20: a web server for annotation and identification of enriched pathways and diseases. Nucleic Acids Res 2011;39:W316–22. 21715386 10.1093/nar/gkr483 PMC3125809

[63] Chen X, Li P, Liu M et al. Gut dysbiosis induces the development of pre-eclampsia through bacterial translocation. Gut 2020;69:513–22. 31900289 10.1136/gutjnl-2019-319101

[64] Abot A, Wemelle E, Laurens C et al. Identification of new enterosynes using prebiotics: roles of bioactive lipids and mu-opioid receptor signalling in humans and mice. Gut 2021;70:1078–87. 33020209 10.1136/gutjnl-2019-320230 PMC8108281

[65] Chevalier C, Stojanović O, Colin DJ et al. Gut microbiota orchestrates energy homeostasis during cold. Cell 2015;163:1360–74. 26638070 10.1016/j.cell.2015.11.004

[66] Lucchini FC, Wueest S, Challa TD et al. ASK1 inhibits browning of white adipose tissue in obesity. Nat Commun 2020;11:1642. 32242025 10.1038/s41467-020-15483-7 PMC7118089

[67] Bath E, Bowden S, Peters C et al. Sperm and sex peptide stimulate aggression in female *Drosophila*. Nat Ecol Evolut 2017;1:0154. 10.1038/s41559-017-0154 PMC5447820 28580431

