# Supplementary material for: Abnormal adipose tissue-derived microbes drive metabolic disorder and exacerbate postnatal growth retardation in piglet
Source: Life Metab. 2024 Jan 17;3(2):load052. doi: 10.1093/lifemeta/load052 (PMC11749387; doi:10.1093/lifemeta/load052)
Supplement: load052_suppl_Supplementary_Figures_S1-S11_Tables_S1-S6 [file load052_suppl_Supplementary_Figures_S1-S11_Tables_S1-S6.docx]

**Supplementary material** **Abnormal adipose tissue-derived microbes drive metabolic disorder and exacerbate postnatal growth retardation in piglet**

Tongxing Song^1,‡^, Ming Qi^2,3,‡^, Yucheng Zhu^1,‡^, Nan Wang^2,3^, Zhibo Liu^1^, Na Li^4,5^, Jiacheng Yang^1^, Yanxu Han^1^, Jing Wang^2,3^, Shiyu Tao^1^, Zhuqing Ren^1^, Yulong Yin^2,3^, Jinshui Zheng^4,5,*^, Bie Tan^2,3,*^

**
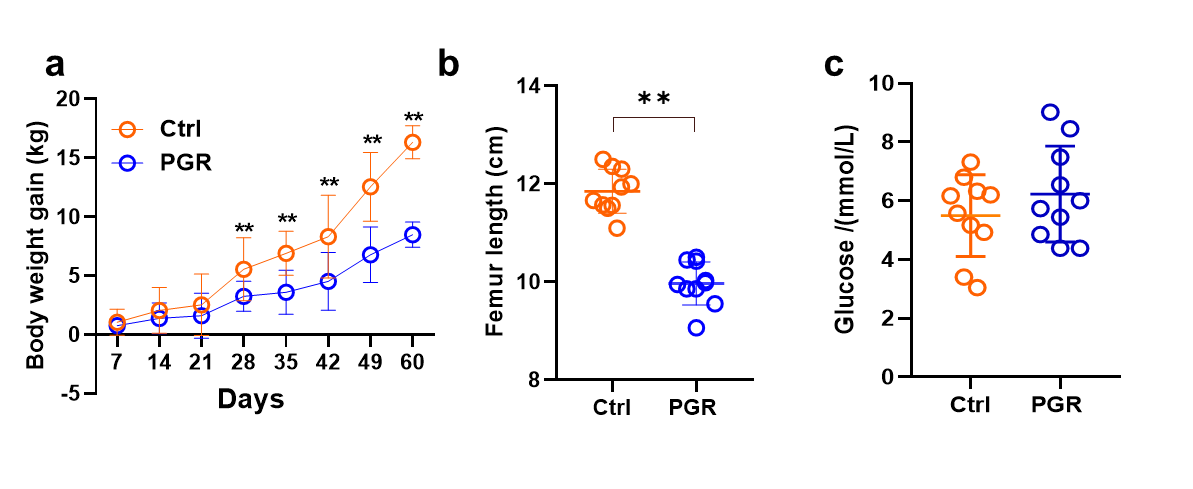
**

**Supplementary Fig S1** Growth and metabolic performance of piglets in the Ctrl and PGR piglets. (a) Body weight gain of the Ctrl and PGR piglets. (b) Femur length of the Ctrl and PGR piglets. (c) Level of glucose in the serum of the Ctrl and PGR pigs. The statistical significance is denoted as: ^*^*P* < 0.05, ^**^*P* < 0.01.


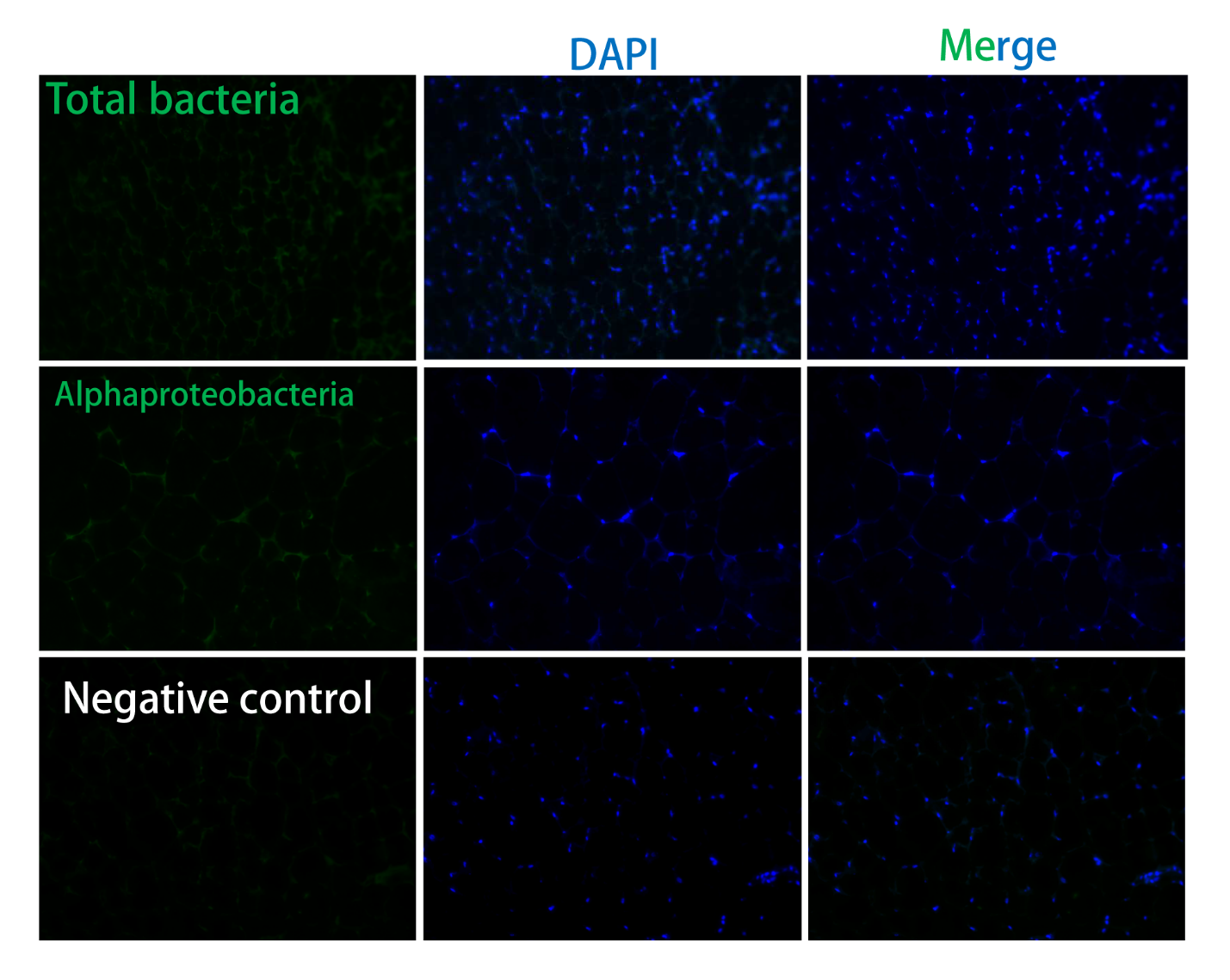


**Supplementary Fig S2** CARD-FISH for adipose tissue from germ free mice. CARD-FISH analysis with total bacteria and *alphaproteobacteria* probes on adipose tissue section from germ-free mice. No probe serves as negative control. The magnification is 200.


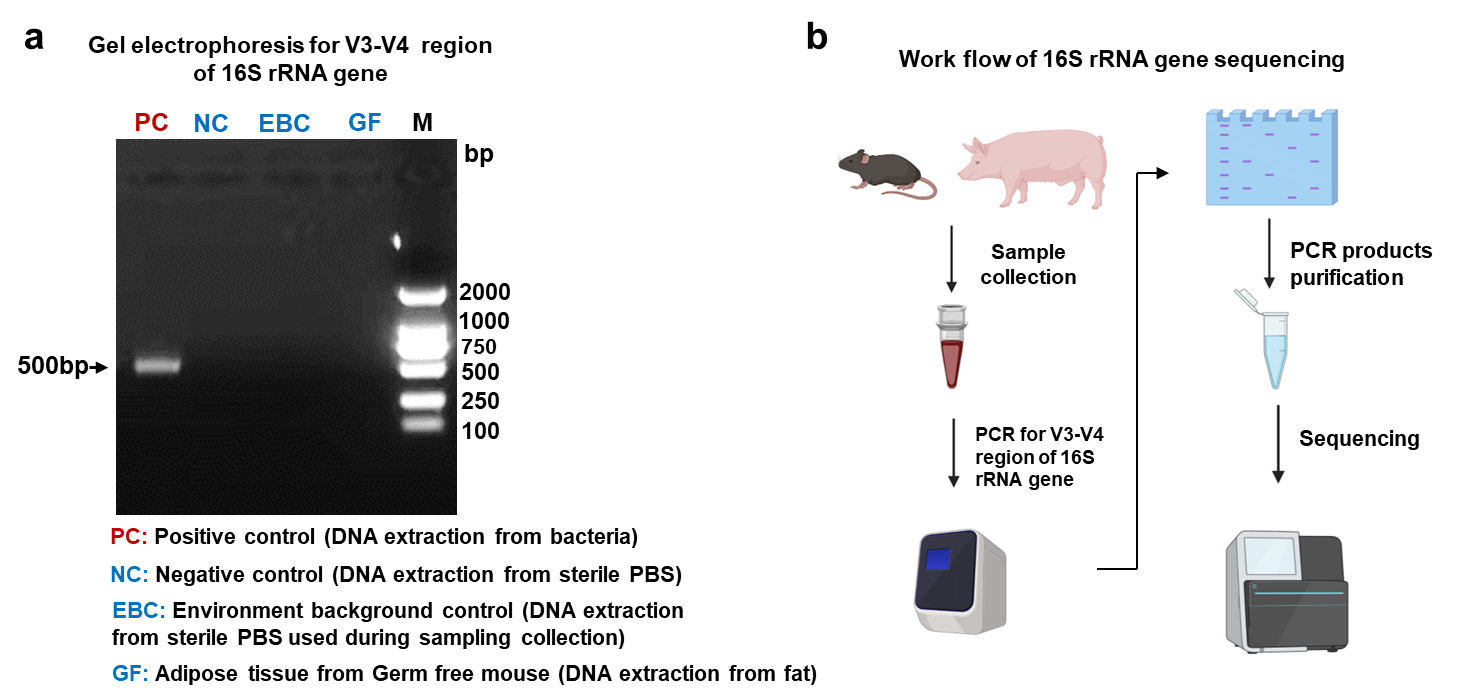


**Supplementary Fig S3** PCR detection for the V3–V4 region of 16S rRNA gene. (a) Work flow of 16S rRNA gene sequencing. (b) Gel electrophoresis for V3–V4 region of 16S rRNA gene. PC: positive control (DNA extraction from bacteria); NC: negative control (DNA extraction from sterile PBS); EBC: environment background control (DNA extraction from sterile PBS used during sampling collection); GF: Adipose tissue from germ free mouse (DNA extraction from fat).


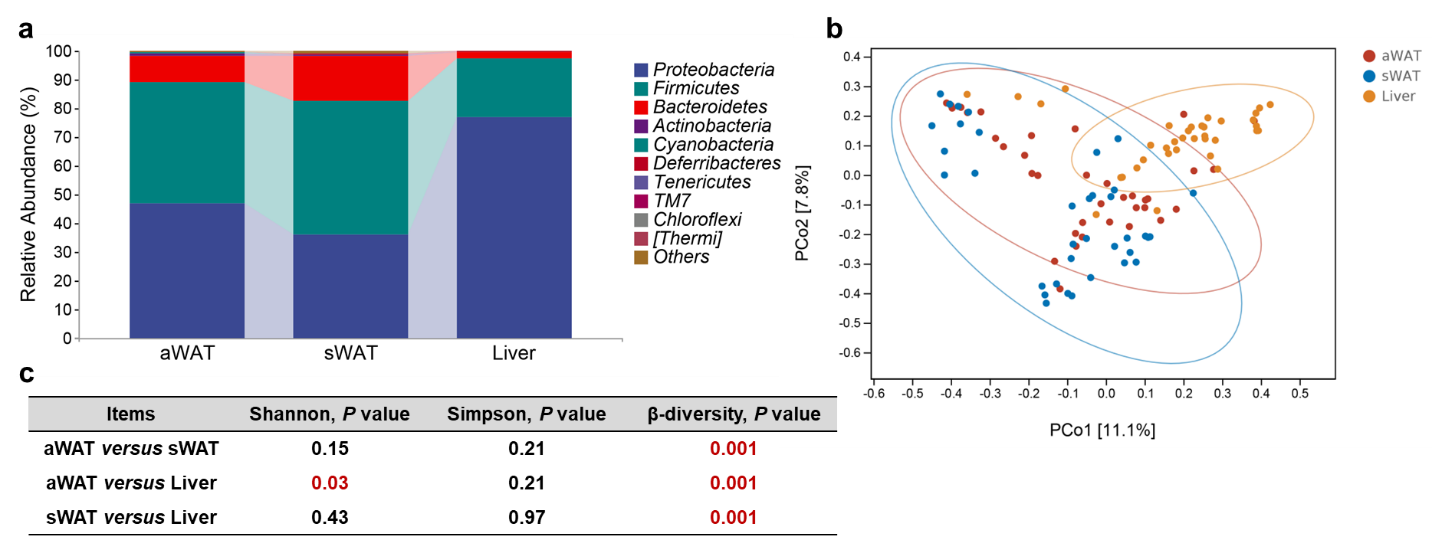


**Supplementary Fig S4** Bacterial distribution across pig tissues. (a) Relative abundance of phyla in sWAT, aWAT, and liver from all piglets. (b) β-diversity analysis for sWAT, aWAT, and liver from all piglets. (c) *P* values for α-diversity (Shannon and Simpson index) and β-diversity analysis.

**
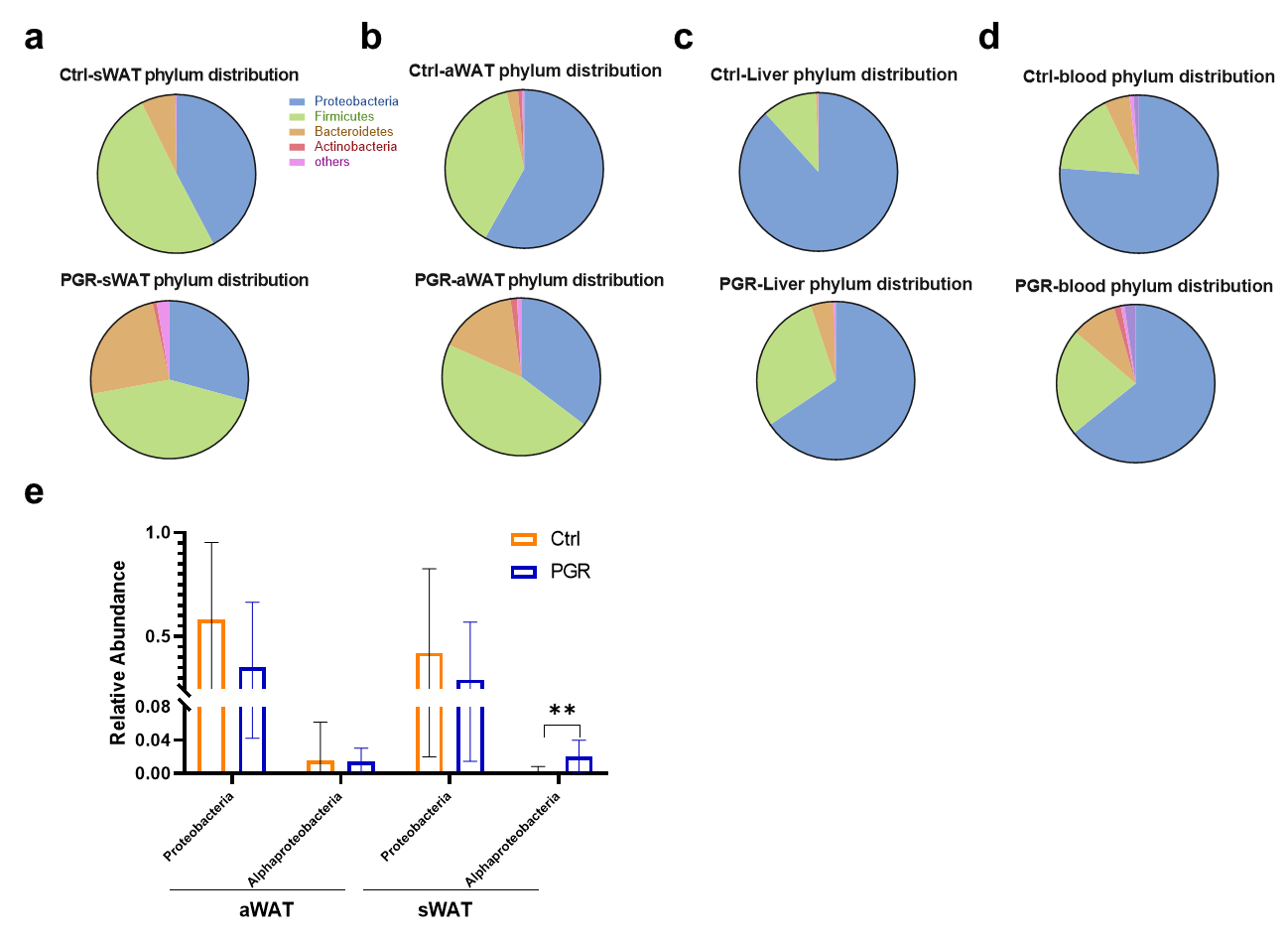
**

**Supplementary Fig S5** Distribution of microbes in sWAT, aWAT, liver, and blood in the Ctrl and PGR groups at phylum level. (a) Relative abundance of phyla in sWAT in the Ctrl and PGR groups. (b) Relative abundance of phyla in aWAT in the Ctrl and PGR groups. (c) Relative abundance of phyla in liver in the Ctrl and PGR groups. (d) Relative abundance of phyla in blood in the Ctrl and PGR groups. (e) Comparison of relative abundance of *Proteobacteria* and *Alphaproteobacteria* in aWAT and sWAT.

**
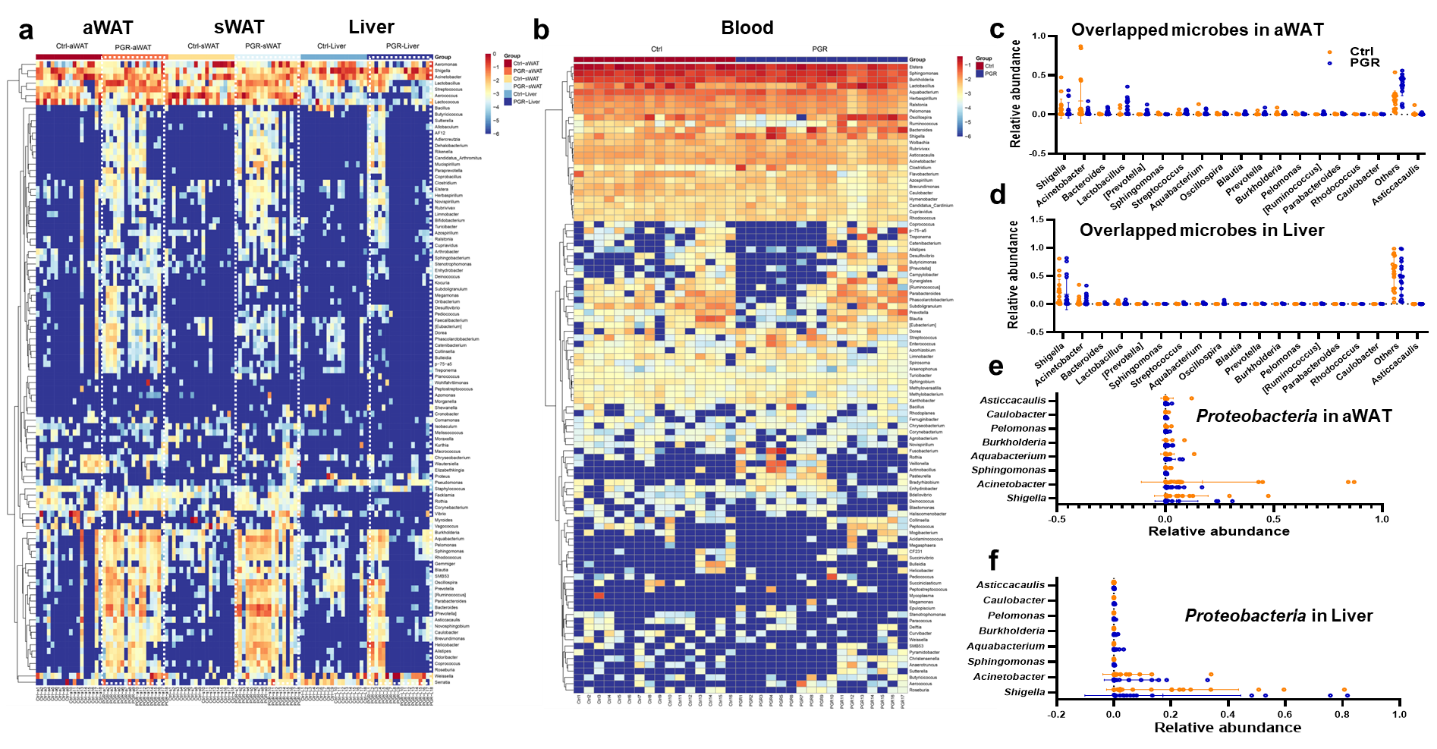
**

**Supplementary Fig S6** Distribution of microbes in aWAT, sWAT, liver, and blood. (a and b) Heatmaps of top 100 microbes in sWAT, aWAT, and liver (a) and blood (b) of the Ctrl and PGR piglets. (c and d) Top 20 genera of aWAT (c) and liver (d). (e and f) Relative abundance of genera from *Proteobacteria* in aWAT (e) and liver (f). The statistical significance is denoted as: ^*^*P* < 0.05, ^**^*P* < 0.01.

**
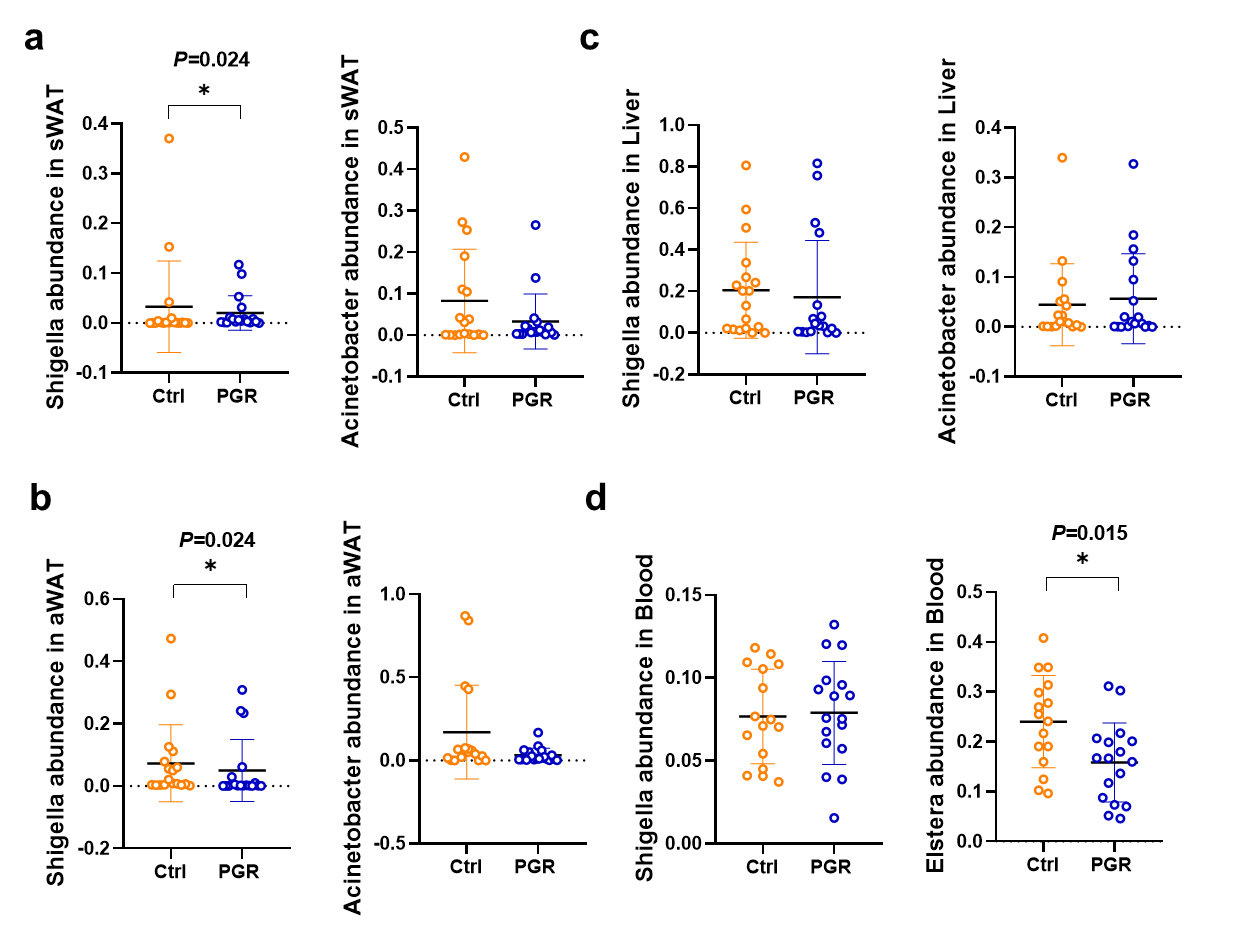
**

**Supplementary Fig S7** Distribution of overlapped top three microbes in sWAT, aWAT, liver, and blood. (a–d) Relative abundance of the top three microbes in sWAT (a), aWAT (b), liver (c), and blood (d). The statistical significance is denoted as: ^*^*P* < 0.05, ^**^*P* < 0.01.

**
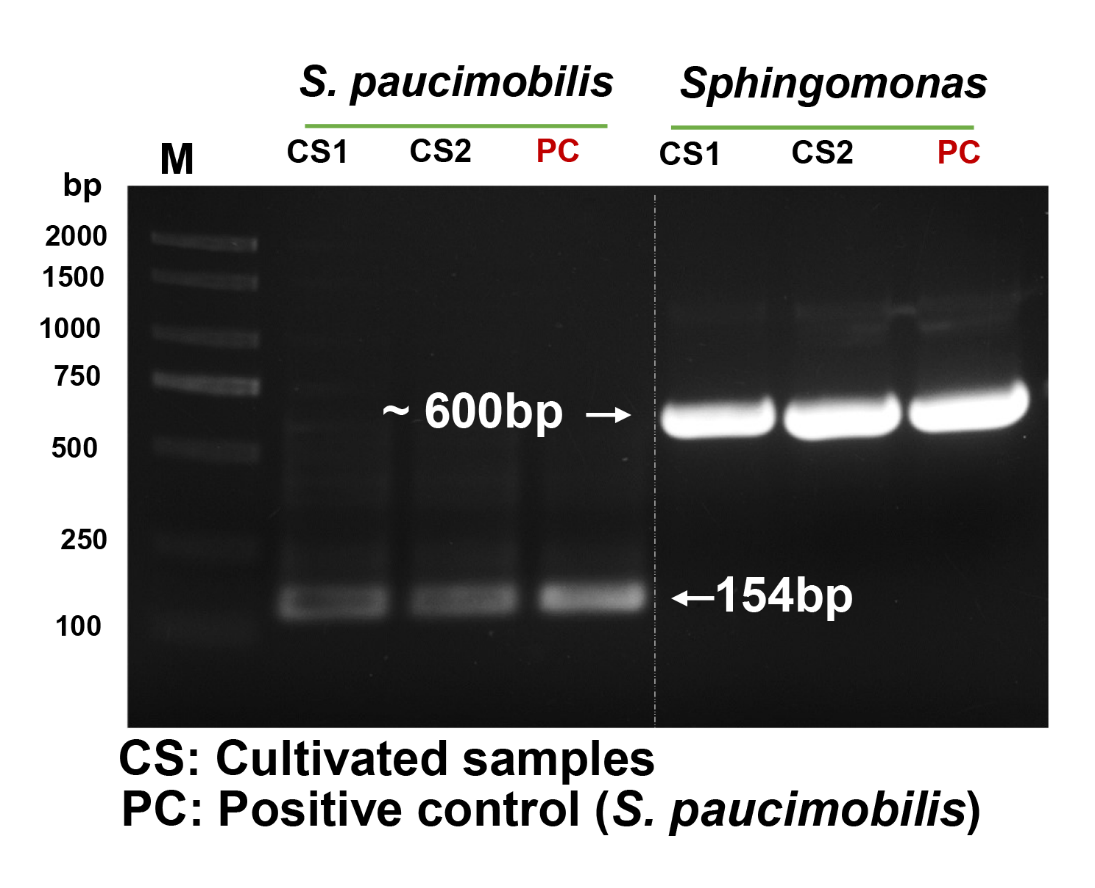
**

**Supplementary Fig S8** PCR detection for *Sphingomonas* and *S. paucimobilis*. Gel electrophoresis for specific DNA fragment of 16S rRNA gene in *Sphingomonas* and *S. paucimobilis*. PC: positive control (*S. paucimobilis*); CS: cultivated samples.

**
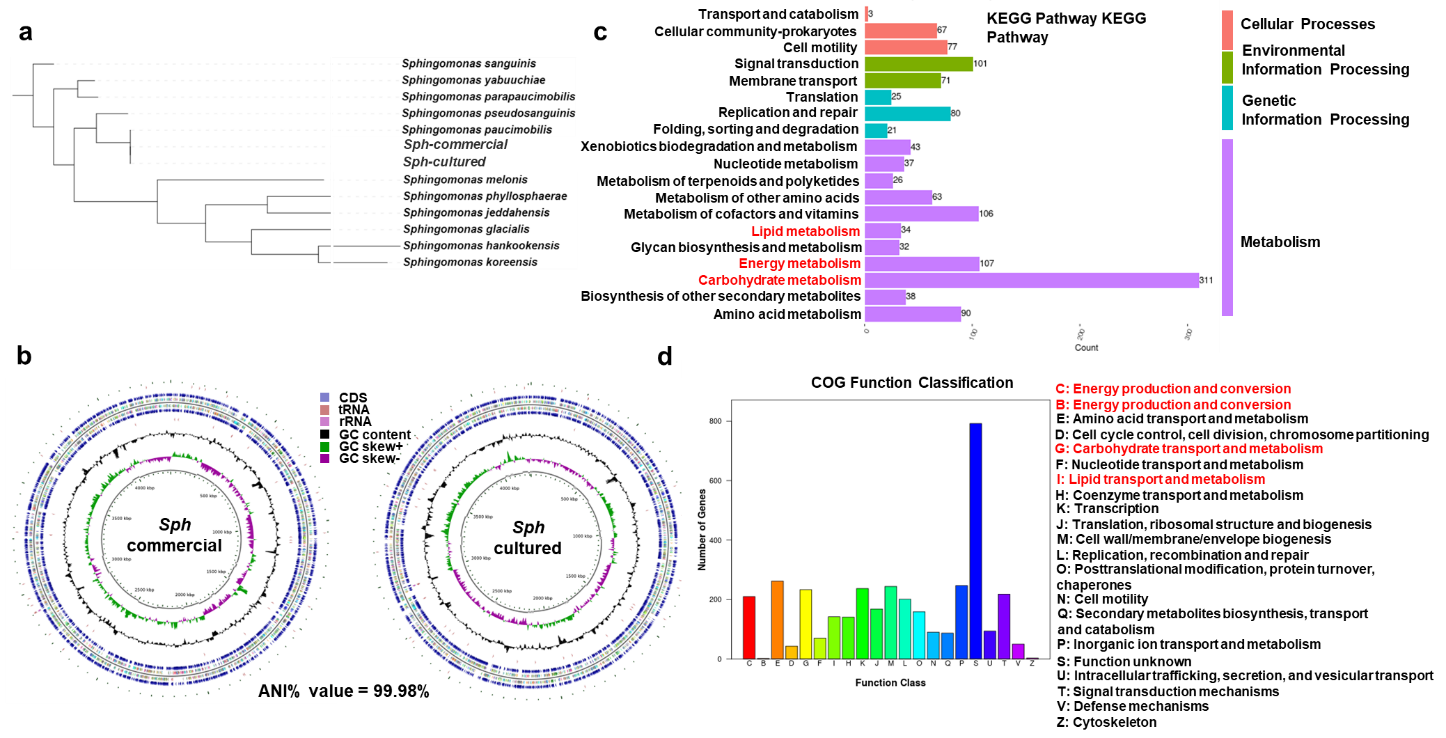
**

**Supplementary Fig S9** Comparative genomic and functional analysis of cultivated *S. paucimobilis* isolates and commercial *S. paucimobilis* (ATCC 29837). (**a**) Phylogenetic analysis of *S. paucimobilis* isolated from sWAT and commercial *S. paucimobilis* based on whole genome sequences. (b) Circular representation of *S. paucimobilis* isolated from sWAT and commercial *S. paucimobilis* based on CDS, tRNA, and rRNA, GO, GC content, GC skew+, and GC skew– results. (c) KEGG analysis of the *S. paucimobilis* genome annotation. (d) COG distribution of the *S. paucimobilis* genome annotation. The statistical significance is denoted as: ^*^*P* < 0.05, ^**^*P* < 0.01.

**
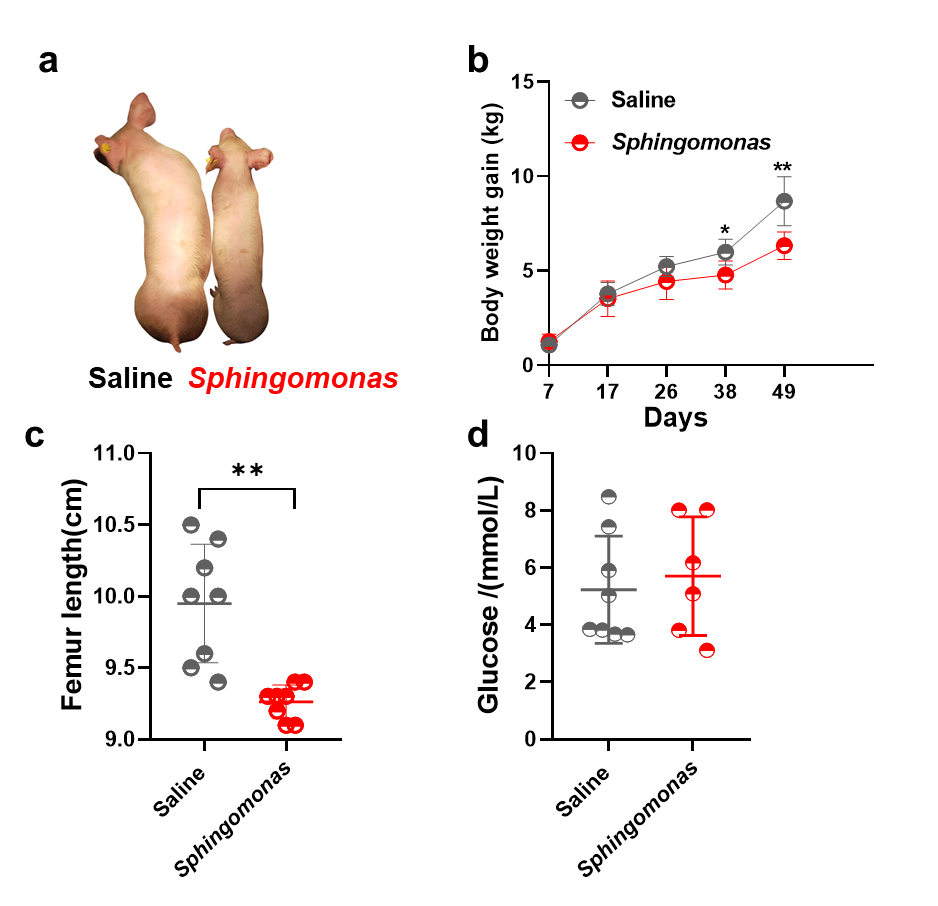
**

**Supplementary Fig S10** Growth performance and metabolic index of piglets in Saline and *Sphingomonas*-gavaged piglets. (a) Body size of piglets. (b) Body weight gain of piglets. (c) Femur length of Saline and *Sphingomonas*-gavaged piglets. (d) Level of glucose in the serum of Saline and *Sphingomonas*-gavaged piglets. The statistical significance is denoted as: ^*^*P* < 0.05, ^**^*P* < 0.01.

**
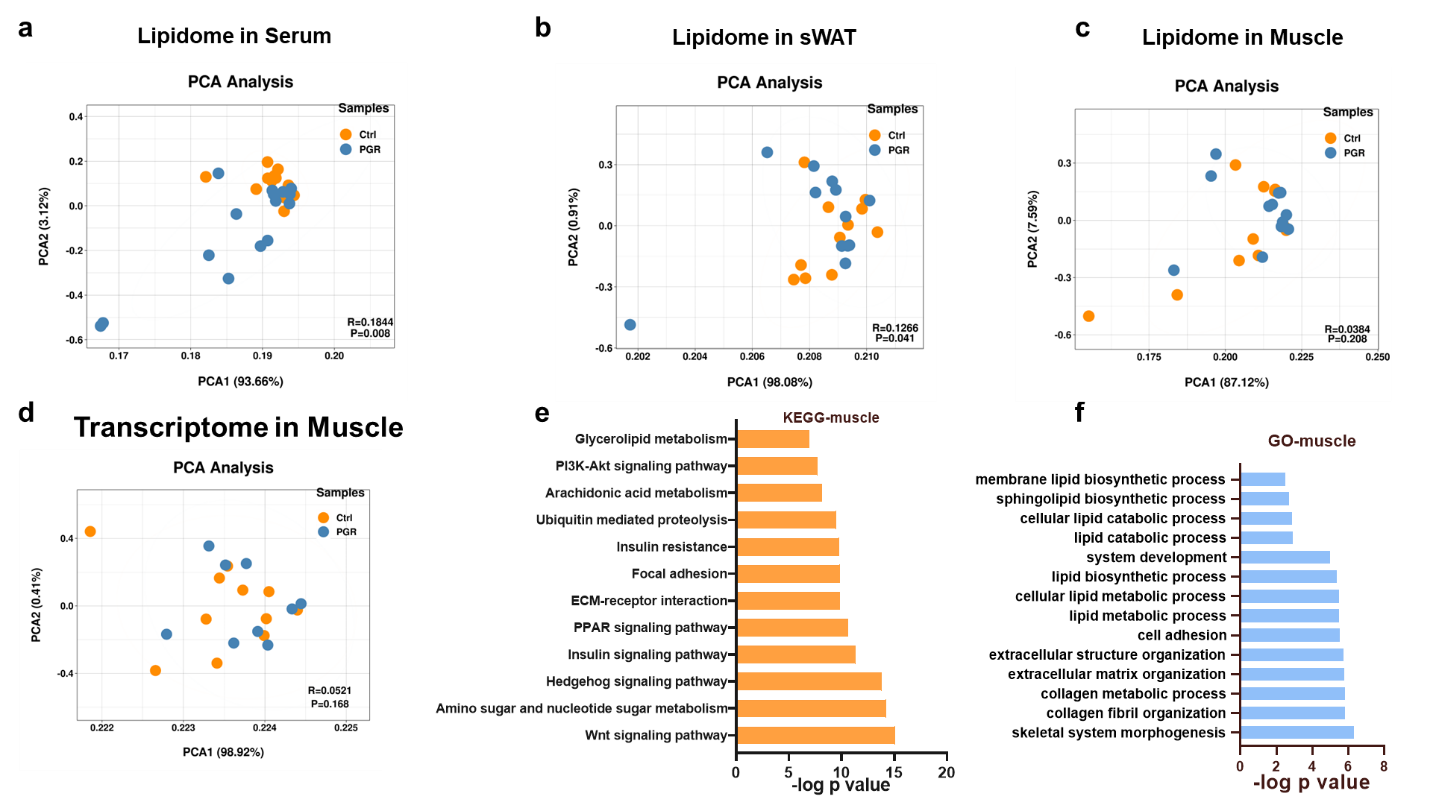
**

**Supplementary Fig S11** Transcriptome and lipidomics signatures of muscle in the Ctrl and PGR groups. (a) Comparison of transcriptome in muscle between the Ctrl and PGR pigs, revealed by PCA (Adonis *P* = 0.168). (b) KEGG analysis of different expression genes (DEGs) in muscle. (c) GO analysis for DEGs in muscle. (d–f) Comparison of lipidomics in serum (d), sWAT (e), and Muscle (f) between the Ctrl and PGR groups revealed by PCA.

**Supplementary Table S1** Probes used in CARD-FISH.

| **Probes target** | **Name** | **Sequence (5’–3’)** | **Reference** |
| --- | --- | --- | --- |
| Total bacteria | EUB338I | GCTGCCTCCCGTAGGAGT | [45] |
|  | EUB338II | GCAGCCACCCGTAGGTGT |  |
|  | EUB338III | GCTGCCACCCGTAGGTGT |  |
| *Alphaproteobacteria* | α-pro | GAATTTCACCTCTACACT | [29] |

**Supplementary Table S2** Primers for PCR Analysis.

| **Species** | **Gene** | **Forward primer (5’–3’)** | **Reverse primer (5’–3’)** | **Reference** |
| --- | --- | --- | --- | --- |
| Pig | *PPARγ* | TGTGGACCTGTCGGTGATG | TGGAGTGGAAATGCTGGAGA | [54] |
|  | *C/EBPα* | CGGTGCGTCTAAGATGAGG | AGCGGTGAGTTTGCGTTT |  |
|  | *ADIPOQ* | CTGGCGAGAAGAGTGAGA | TGCTGAACGGTAGACATAGGC |  |
|  | *FABP4* | GGAAGGTGGCTGGCATGGC | CCTCCATCTAAGGTTATGGTGCTCTTG |  |
|  | *HSL* | ACCCTCGGCTGTCAACTTCTT | ACTTTCTCCTCCTTGGTGCTAATCT |  |
|  | *ATGL* | GCCCACGAGTGATAGCATCC | CAGCAGGTTGGACAGGGTG |  |
|  | *GLUT4* | GAAGGAAGAAGGCAATGCTG | GAGGAACCGTCCAAGAATGA |  |
|  | *LEPTIN* | AGATCCTCACCAGTCTGCCTTCC | CCAGGCTCTCCAAGGTCTCCAG |  |
|  | *IL-1β* | CAGCCATGGCCATAGTACCT | CCACGATGACAGACACCATC | [29] |
|  | *IL-6* | GGCAAAAGGGAAAGAATCCAG | CGTTCTGTGACTGCAGCTTATCC |  |
|  | *IL-8* | GCTCTCTGTGAGGCTGCAGTTC | AAGGTGTGGAATGCGTATTTATGC |  |
|  | *TNF-α* | ACAGGCCAGCTCCCTCTTAT | CCTCGCCCTCCTGAATAAAT |  |
|  | *IFNγ* | TTCAGCTTTGCGTGACTTTG | GGTCCACCATTAGGTACATCTG |  |
|  | *NFkB* | AGCCATTGACGTGATCCAGG | CGAAATCGTGGGGCACTTTG |  |
|  | *IL10* | GGGCTATTTGTCCTGACTGC | GGGCTCCCTAGTTTCTCTTCC |  |
|  | *ADPGK* | ATTTTCGGGCCTCCGTCTTT | GCGGAGCTGAGAACAGAGAA | This manuscript |
|  | *HK3* | TGGGACCCTCTACAAGCTACA | TTGCCAGACCCGTCCTTT |  |
|  | *ACACA* | TGGAGGAGAAGGAGGG | GGTGCAAGCCAGACAT |  |
|  | *ACSL4* | AAGCACGAACAATAGACA | GACAGAGCGATATGGAC |  |
|  | *FASN* | CTCCAAGCAGGCGAACACG | CCACGAAGGGAAGCAGGGT |  |
|  | *GPD2* | CAAATGGATGAAAACACGCTT | TCCACAACTTCGGTCTACAGG |  |
|  | *AGPAT1* | CCTTCTACAACGGCTGGAT | GCTGTGAGGGAGGGAAGTGG |  |
|  | *CDS2* | ATGACGGAACTAAGGCAGAGG | CACTGTCGGATGCAGTCTCT |  |
|  | *β-actin* | CTGCGGCATCCACGAAACT | AGGGCCGTGATCTCCTTCTG |  |
|  | *18s* | CCCACGGAATCGAGAAAGAG | TTGACGGAAGGGCACCA |  |
| Bacteria | *Sphingomonas-600* | MRGWCCAAAGATTTATCG | CMAADCACCAWGTGMCCKGA | [36] |
|  | *Sphingomonas-154* | AAGCCGACGATCCATAGCTG | ACCCTAGGGCCTTCATCACT | This manuscript |
|  | *Sphingomonas-207* | CATGCAAGTCGAACGAAGGC | CAGCTATGGATCGTCGGCTT |  |

**Supplementary Table S3** Top 100 microbes of genus level in blood.

| **Bacteria** | **Mean Ctrl** | **Mean PGR** | ***P* value** |
| --- | --- | --- | --- |
| *Elstera* | 0.240258 | 0.1585 | 0.010235 |
| *Sphingomonas* | 0.126654 | 0.100789 | 0.136923 |
| *Burkholderia* | 0.076692 | 0.078903 | 0.832983 |
| *Lactobacillus* | 0.062289 | 0.056169 | 0.815317 |
| *Aquabacterium* | 0.041126 | 0.032151 | 0.1191 |
| *Oscillospira* | 0.023693 | 0.038273 | 0.350265 |
| *Bacteroides* | 0.005937 | 0.04131 | 0.015881 |
| *Shigella* | 0.012125 | 0.030885 | 0.104732 |
| *Herbaspirillum* | 0.020349 | 0.017684 | 0.314868 |
| *Ralstonia* | 0.012527 | 0.012953 | 0.786925 |
| *Pelomonas* | 0.012524 | 0.012086 | 0.811018 |
| *Asticcacaulis* | 0.009517 | 0.009354 | 0.897811 |
| *Acinetobacter* | 0.008209 | 0.009501 | 0.294431 |
| *Ruminococcus* | 0.005359 | 0.008529 | 0.434198 |
| *Rubrivivax* | 0.006822 | 0.006733 | 0.925224 |
| *Flavobacterium* | 0.009363 | 0.00404 | 0.057989 |
| *Wolbachia* | 0.005749 | 0.007073 | 0.376412 |
| *Clostridium* | 0.001294 | 0.010864 | 0.137542 |
| *Subdoligranulum* | 0.008806 | 0.003732 | 0.060451 |
| *Fusobacterium* | 0.000179 | 0.011751 | 0.163226 |
| *Blautia* | 0.008392 | 0.002847 | 0.185796 |
| *Parabacteroides* | 0.004327 | 0.006052 | 0.529252 |
| *Synergistes* | 0.001391 | 0.007999 | 0.141691 |
| *p-75-a5* | 0.001376 | 0.007682 | 0.187267 |
| *Phascolarctobacterium* | 0.00336 | 0.005747 | 0.245711 |
| *Brevundimonas* | 0.006077 | 0.003067 | 0.002753 |
| *Caulobacter* | 0.004989 | 0.002569 | 0.001141 |
| *Azospirillum* | 0.003404 | 0.003865 | 0.556875 |
| *Prevotella* | 0.00286 | 0.004085 | 0.647158 |
| *Streptococcus* | 0.001818 | 0.004791 | 0.191252 |
| *Candidatus_Cardinium* | 0.002504 | 0.003501 | 0.167502 |
| *Dorea* | 0.002089 | 0.003627 | 0.31134 |
| *Cupriavidus* | 0.002784 | 0.002731 | 0.909981 |
| *Rhodococcus* | 0.002667 | 0.002775 | 0.790137 |
| *Enterococcus* | 0.000684 | 0.004601 | 0.035729 |
| *Veillonella* | 2.77E-05 | 0.004991 | 0.116938 |
| *Hymenobacter* | 0.002978 | 0.002212 | 0.315827 |
| *[Ruminococcus]* | 0.002187 | 0.00279 | 0.687817 |
| *Actinobacillus* | 0.000132 | 0.004679 | 0.090849 |
| *Mycoplasma* | 0.003927 | 0 | 0.310141 |
| *Limnobacter* | 0.001958 | 0.001616 | 0.628096 |
| *Pediococcus* | 6.68E-05 | 0.003305 | 0.335857 |
| *Treponema* | 0.000655 | 0.0027 | 0.368507 |
| *Desulfovibrio* | 0.000627 | 0.002562 | 0.073553 |
| *Bacillus* | 0.00065 | 0.002418 | 0.400783 |
| *[Eubacterium]* | 0.001966 | 0.001097 | 0.212198 |
| *Methylobacterium* | 0.001108 | 0.001394 | 0.395143 |
| *Sphingobium* | 0.001102 | 0.001253 | 0.455314 |
| *Xanthobacter* | 0.001103 | 0.001248 | 0.653174 |
| *[Prevotella]* | 0.001001 | 0.001135 | 0.906393 |
| *Aerococcus* | 5.27E-05 | 0.001833 | 0.324532 |
| *Collinsella* | 0.001054 | 0.000777 | 0.715808 |
| *Campylobacter* | 0.000312 | 0.001353 | 0.353172 |
| *Methyloversatilis* | 0.000827 | 0.000856 | 0.889716 |
| *Catenibacterium* | 0.000705 | 0.000908 | 0.762062 |
| *Bulleidia* | 0.001594 | 2.33E-05 | 0.115441 |
| *Arsenophonus* | 0.000642 | 0.000889 | 0.334688 |
| *Butyricimonas* | 0.000395 | 0.00111 | 0.135349 |
| *Spirosoma* | 0.000851 | 0.000665 | 0.40492 |
| *Peptococcus* | 0.000467 | 0.001002 | 0.320707 |
| *Pasteurella* | 0 | 0.001378 | 0.045003 |
| *Azorhizobium* | 0.000585 | 0.000795 | 0.437668 |
| *Turicibacter* | 0.000627 | 0.000532 | 0.083021 |
| *Enhydrobacter* | 0.000368 | 0.000763 | 0.321357 |
| *Mogibacterium* | 0.000276 | 0.000846 | 0.240802 |
| *Acidaminococcus* | 0.000362 | 0.000723 | 0.579522 |
| *Bradyrhizobium* | 0.000405 | 0.000626 | 0.223776 |
| *Rothia* | 0.000111 | 0.000819 | 0.10378 |
| *Ferruginibacter* | 0.000345 | 0.000558 | 0.238952 |
| *Succiniclasticum* | 0.000859 | 5.88E-05 | 0.151708 |
| *Paracoccus* | 0.00017 | 0.000695 | 0.360307 |
| *Haliscomenobacter* | 0.000541 | 0.000201 | 0.059183 |
| *Deinococcus* | 0.000266 | 0.000417 | 0.495836 |
| *Delftia* | 0.000247 | 0.00043 | 0.411523 |
| *Stenotrophomonas* | 0.000109 | 0.000546 | 0.385968 |
| *Pyramidobacter* | 0.000129 | 0.000527 | 0.311334 |
| *Epulopiscium* | 0.000354 | 0.0003 | 0.754383 |

**Supplementary Table S4.** Top 100 microbes of genus level in aWAT

| **Bacteria** | **Mean Ctrl** | **Mean PGR** | ***P* value** |
| --- | --- | --- | --- |
| *Shigella* | 0.073165 | 0.050526 | 0.548698 |
| *Aeromonas* | 0.070463 | 0.046116 | 0.61034 |
| *Acinetobacter* | 0.170773 | 0.031794 | 0.046603 |
| *Aerococcus* | 0.039121 | 0.009358 | 0.086018 |
| *Lactococcus* | 0.143425 | 0.04032 | 0.097349 |
| *Weissella* | 0.018013 | 0.039791 | 0.489419 |
| *Lactobacillus* | 0.011645 | 0.096191 | 0.001929 |
| *Vibrio* | 0.088994 | 0.009837 | 0.157636 |
| *Serratia* | 0.041943 | 0.025601 | 0.696927 |
| *Bacteroides* | 0.001179 | 0.022089 | 0.010655 |
| *Myroides* | 0.007533 | 8.78E-05 | 0.116509 |
| *Streptococcus* | 0.010536 | 0.030196 | 0.005196 |
| *[Prevotella]* | 0.000201 | 0.01978 | 0.018588 |
| *Aquabacterium* | 0.008973 | 0.018702 | 0.318961 |
| *Vagococcus* | 0.002437 | 0.000624 | 0.159502 |
| *Oscillospira* | 0.001575 | 0.009218 | 0.006546 |
| *Helicobacter* | 0.000179 | 0.003398 | 0.015287 |
| *Wautersiella* | 0.003137 | 0.001519 | 0.273707 |
| *Proteus* | 0.00014 | 0.000379 | 0.527622 |
| *Prevotella* | 0.00294 | 0.010644 | 0.178523 |
| *Burkholderia* | 0.007052 | 0.00727 | 0.968828 |
| *Blautia* | 0.000926 | 0.010683 | 0.006057 |
| *Wohlfahrtiimonas* | 3.03E-06 | 0.017808 | 0.324456 |
| *Alistipes* | 2.19E-05 | 0.00157 | 0.00447 |
| *Coprococcus* | 0.000111 | 0.005888 | 0.044501 |
| *[Ruminococcus]* | 0.000371 | 0.008309 | 0.017189 |
| *Bacillus* | 1.95E-05 | 0.002004 | 0.268974 |
| *Asticcacaulis* | 0.007334 | 0.002872 | 0.520062 |
| *Sphingomonas* | 0.002793 | 0.002609 | 0.921911 |
| *Pelomonas* | 0.002034 | 0.003587 | 0.420652 |
| *Odoribacter* | 4.88E-05 | 0.001449 | 0.001476 |
| *SMB53* | 0.001574 | 0.005919 | 0.100942 |
| *Parabacteroides* | 0.000853 | 0.004209 | 0.029586 |
| *Caulobacter* | 0.000978 | 0.001769 | 0.396279 |
| *Paraprevotella* | 0 | 0.003185 | 0.003903 |
| *Rhodococcus* | 0.000791 | 0.001676 | 0.12976 |
| *Mucispirillum* | 0 | 0.000303 | 0.006913 |
| *Rothia* | 0.002008 | 0.002173 | 0.88337 |
| *Corynebacterium* | 0.001305 | 0.002431 | 0.349387 |
| *Novosphingobium* | 0.000126 | 0.001196 | 0.002882 |
| *Roseburia* | 6.27E-05 | 0.002179 | 0.018901 |
| *Brevundimonas* | 0.000384 | 0.001428 | 0.012942 |
| *Facklamia* | 0.000813 | 0.00102 | 0.680159 |
| *Staphylococcus* | 0.001421 | 0.000446 | 0.013948 |
| *[Eubacterium]* | 0.000802 | 0.002125 | 0.21875 |
| *Elstera* | 0.00104 | 0.000563 | 0.491449 |
| *Kurthia* | 3.21E-05 | 0.000541 | 0.163449 |
| *Phascolarctobacterium* | 0.000542 | 0.002262 | 0.059747 |
| *Gemmiger* | 0.000226 | 0.001889 | 0.013461 |
| *Chryseobacterium* | 0.001146 | 0.00042 | 0.120302 |
| *Clostridium* | 0.00041 | 0.00101 | 0.19918 |
| *Rikenella* | 0 | 0.000925 | 0.002841 |
| *Pseudomonas* | 0.000133 | 5.45E-05 | 0.210323 |
| *Butyricicoccus* | 0.00055 | 0.000633 | 0.887363 |
| *Elizabethkingia* | 0.001494 | 0.000361 | 0.081755 |
| *Ralstonia* | 0.000701 | 0.000729 | 0.961959 |
| *Herbaspirillum* | 0.000721 | 0.000613 | 0.85888 |
| *Shewanella* | 3.03E-06 | 0.000227 | 0.330827 |
| *Cronobacter* | 8.39E-05 | 1.78E-05 | 0.305029 |
| *Sutterella* | 4.53E-05 | 0.000742 | 0.00568 |
| *Candidatus_Arthromitus* | 0 | 0.000721 | 0.005355 |
| *Dorea* | 0.000283 | 0.00114 | 0.088076 |
| *Dehalobacterium* | 0 | 0.00052 | 0.005998 |
| *Collinsella* | 0.000396 | 0.001194 | 0.215458 |
| *Azomonas* | 0 | 5.42E-06 | 0.020536 |
| *Bifidobacterium* | 0.000138 | 0.000482 | 0.144339 |
| *Novispirillum* | 0.000217 | 0.000163 | 0.722285 |
| *Subdoligranulum* | 9.65E-05 | 0.001113 | 0.019611 |
| *Comamonas* | 0.000271 | 0.00075 | 0.280689 |
| *Coprobacillus* | 1.04E-05 | 0.000492 | 0.0038 |
| *Azospirillum* | 0.000252 | 0.00031 | 0.821697 |
| *Megamonas* | 4.71E-05 | 0.001001 | 0.132959 |
| *Faecalibacterium* | 0.000296 | 0.000451 | 0.599089 |
| *p-75-a5* | 0.000397 | 0.000701 | 0.513112 |
| *Morganella* | 1.66E-05 | 4.96E-06 | 0.506877 |
| *Allobaculum* | 3.62E-05 | 0.00065 | 0.019115 |
| *Bulleidia* | 0.000284 | 0.000733 | 0.270024 |

**Supplementary Table S5.** Top 100 microbes of genus level in sWAT

| **Bacteria** | **Mean Ctrl** | **Mean PGR** | ***P* value** |
| --- | --- | --- | --- |
| *Shigella* | 0.032757 | 0.02021 | 0.591001 |
| *Aeromonas* | 0.241847 | 0.078211 | 0.07991 |
| *Acinetobacter* | 0.08268 | 0.03311 | 0.145495 |
| *Aerococcus* | 0.202792 | 0.138872 | 0.457773 |
| *Lactococcus* | 0.086085 | 0.03492 | 0.21792 |
| *Weissella* | 0.000323 | 0.009598 | 0.135514 |
| *Lactobacillus* | 0.003793 | 0.039844 | 0.000926 |
| *Vibrio* | 0.001307 | 0.009051 | 0.100277 |
| *Serratia* | 0.000288 | 0.011372 | 0.30063 |
| *Bacteroides* | 9.96E-05 | 0.050541 | 0.002301 |
| *Myroides* | 0.067229 | 0.000792 | 0.229327 |
| *Streptococcus* | 0.013688 | 0.008266 | 0.448276 |
| *[Prevotella]* | 8.13E-05 | 0.032377 | 0.000821 |
| *Aquabacterium* | 0.000514 | 0.018742 | 6.84E-05 |
| *Vagococcus* | 0.044782 | 0.002063 | 0.31648 |
| *Oscillospira* | 9.12E-05 | 0.014937 | 6.98E-05 |
| *Helicobacter* | 1.77E-06 | 0.00899 | 0.001853 |
| *Wautersiella* | 0.000175 | 0.03157 | 0.299713 |
| *Proteus* | 0 | 0.000229 | 0.050353 |
| *Prevotella* | 0.000505 | 0.007201 | 0.002112 |
| *Burkholderia* | 0.001527 | 0.006098 | 0.038097 |
| *Blautia* | 0.000568 | 0.010045 | 0.049403 |
| *Wohlfahrtiimonas* | 9.72E-06 | 2.11E-06 | 0.449241 |
| *Alistipes* | 0 | 0.009013 | 0.001283 |
| *Coprococcus* | 5.5E-05 | 0.006774 | 0.019904 |
| *[Ruminococcus]* | 2.87E-05 | 0.003359 | 0.001768 |
| *Bacillus* | 0 | 0.000123 | 0.12699 |
| *Asticcacaulis* | 0.000144 | 0.001851 | 0.000244 |
| *Sphingomonas* | 0.000987 | 0.005024 | 0.014532 |
| *Pelomonas* | 0.00026 | 0.004984 | 0.00035 |
| *Odoribacter* | 0 | 0.00473 | 0.000853 |
| *SMB53* | 8.01E-05 | 0.000685 | 0.001123 |
| *Parabacteroides* | 1.62E-05 | 0.003189 | 0.00075 |
| *Caulobacter* | 3.82E-05 | 0.002871 | 0.000236 |
| *Paraprevotella* | 5.3E-06 | 0.002852 | 0.014773 |
| *Rhodococcus* | 0.000474 | 0.002357 | 0.002519 |
| *Mucispirillum* | 0 | 0.004075 | 0.03156 |
| *Rothia* | 0.000164 | 0.000906 | 0.003131 |
| *Corynebacterium* | 0.000239 | 0.00094 | 0.001113 |
| *Novosphingobium* | 0 | 0.003108 | 0.017863 |
| *Roseburia* | 0.000216 | 0.00178 | 0.026394 |
| *Brevundimonas* | 4.76E-05 | 0.00212 | 0.000138 |
| *Facklamia* | 0.000687 | 0.001385 | 0.365637 |
| *Staphylococcus* | 0.00071 | 0.000375 | 0.50947 |
| *[Eubacterium]* | 0.00021 | 0.000315 | 0.480051 |
| *Elstera* | 0.000603 | 0.000843 | 0.642369 |
| *Kurthia* | 0.000124 | 0.000996 | 0.284904 |
| *Phascolarctobacterium* | 1.08E-05 | 0.000181 | 0.010677 |
| *Gemmiger* | 0.000156 | 0.000475 | 0.093657 |
| *Chryseobacterium* | 0.000303 | 0.000447 | 0.479002 |
| *Clostridium* | 0.000236 | 0.000638 | 0.118013 |
| *Rikenella* | 0 | 0.001102 | 0.002365 |
| *Pseudomonas* | 7.4E-05 | 6.93E-05 | 0.934012 |
| *Butyricicoccus* | 2.77E-05 | 0.000725 | 0.00975 |
| *Elizabethkingia* | 0.000384 | 9.6E-05 | 0.132763 |
| *Ralstonia* | 0.000122 | 0.000487 | 0.034631 |
| *Herbaspirillum* | 0.000158 | 0.000535 | 0.023517 |
| *Shewanella* | 2.46E-05 | 0.000175 | 0.347305 |
| *Cronobacter* | 0.001474 | 0.000253 | 0.412421 |
| *Sutterella* | 0 | 0.001194 | 0.019604 |
| *Candidatus_Arthromitus* | 0 | 0.001144 | 0.028314 |
| *Dorea* | 0.000125 | 0.000336 | 0.171449 |
| *Dehalobacterium* | 0 | 0.000814 | 0.004667 |
| *Collinsella* | 1.28E-05 | 0.00014 | 0.038577 |
| *Azomonas* | 1.76E-06 | 0.001764 | 0.324851 |
| *Bifidobacterium* | 2.99E-05 | 0.000689 | 0.144514 |
| *Novispirillum* | 3.18E-05 | 0.00051 | 0.030893 |
| *Subdoligranulum* | 0 | 0.000222 | 0.048903 |
| *Comamonas* | 0.000271 | 5.31E-05 | 0.376461 |
| *Coprobacillus* | 0 | 0.000767 | 0.03704 |
| *Azospirillum* | 0.000117 | 0.000419 | 0.049261 |
| *Megamonas* | 0 | 0.000167 | 0.216798 |
| *Faecalibacterium* | 0.000124 | 0.000159 | 0.718778 |
| *p-75-a5* | 2.17E-05 | 7.27E-05 | 0.107151 |
| *Morganella* | 0 | 1.86E-05 | 0.324375 |
| *Allobaculum* | 1.04E-05 | 0.000347 | 0.045861 |
| *Bulleidia* | 7.86E-06 | 4.59E-05 | 0.186046 |

**Supplementary Table S6.** Top 100 microbes of genus level in Liver

| **Bacteria** | **Mean Ctrl** | **Mean PGR** | ***P* value** |
| --- | --- | --- | --- |
| *Shigella* | 0.20522 | 0.171535 | 0.691589 |
| *Aeromonas* | 0.067101 | 0.016495 | 0.243104 |
| *Acinetobacter* | 0.044485 | 0.056733 | 0.673252 |
| *Aerococcus* | 0.000239 | 6.7E-05 | 0.076484 |
| *Lactococcus* | 0.052979 | 0.001315 | 0.326883 |
| *Weissella* | 0.022646 | 0.18245 | 0.059828 |
| *Lactobacillus* | 0.009371 | 0.009644 | 0.967051 |
| *Vibrio* | 0.000574 | 0.000652 | 0.916469 |
| *Serratia* | 0.006421 | 0.007503 | 0.866079 |
| *Bacteroides* | 6.62E-05 | 0.003362 | 0.119768 |
| *Myroides* | 3.76E-06 | 5.11E-05 | 0.24667 |
| *Streptococcus* | 0.000867 | 0.004593 | 0.147443 |
| *[Prevotella]* | 4.1E-05 | 0.004354 | 0.079004 |
| *Aquabacterium* | 0.000123 | 0.004935 | 0.029105 |
| *Vagococcus* | 1.06E-05 | 1.17E-05 | 0.946327 |
| *Oscillospira* | 0.000577 | 0.010958 | 0.051623 |
| *Helicobacter* | 1.15E-05 | 0.024226 | 0.087976 |
| *Wautersiella* | 2.74E-05 | 7.29E-05 | 0.269898 |
| *Proteus* | 0.02381 | 0.000531 | 0.306753 |
| *Prevotella* | 0.001843 | 0.001379 | 0.669677 |
| *Burkholderia* | 0.000209 | 0.001835 | 0.068151 |
| *Blautia* | 0.000287 | 5.24E-05 | 0.090655 |
| *Wohlfahrtiimonas* | 0 | 0.00026 | 0.30777 |
| *Alistipes* | 1.28E-05 | 0.006775 | 0.081997 |
| *Coprococcus* | 3.83E-05 | 0.002361 | 0.064133 |
| *[Ruminococcus]* | 0.000297 | 0.002296 | 0.071594 |
| *Bacillus* | 0.008972 | 0.002103 | 0.201376 |
| *Asticcacaulis* | 1.13E-05 | 0.000427 | 0.007768 |
| *Sphingomonas* | 0.00016 | 0.000921 | 0.035367 |
| *Pelomonas* | 4.12E-05 | 0.001353 | 0.021601 |
| *Odoribacter* | 5.3E-06 | 0.004904 | 0.068719 |
| *SMB53* | 0.000719 | 0.000105 | 0.038388 |
| *Parabacteroides* | 0.000146 | 0.000375 | 0.237372 |
| *Caulobacter* | 4.45E-06 | 0.000767 | 0.011706 |
| *Paraprevotella* | 0 | 0.000306 | 0.070596 |
| *Rhodococcus* | 0.000299 | 0.000694 | 0.236345 |
| *Mucispirillum* | 4.22E-06 | 0.001644 | 0.173355 |
| *Rothia* | 0 | 1.48E-05 | 0.061985 |
| *Corynebacterium* | 4.45E-05 | 3.34E-05 | 0.675643 |
| *Novosphingobium* | 0 | 0.000395 | 0.021626 |
| *Roseburia* | 5.39E-05 | 0.000369 | 0.096878 |
| *Brevundimonas* | 0 | 0.000594 | 0.033204 |
| *Facklamia* | 0 | 0 | #DIV/0! |
| *Staphylococcus* | 0.000119 | 0.000725 | 0.100541 |
| *[Eubacterium]* | 0.000106 | 0 | 0.029825 |
| *Elstera* | 3.36E-05 | 0.000225 | 0.120543 |
| *Kurthia* | 0.001482 | 2.09E-05 | 0.331147 |
| *Phascolarctobacterium* | 0.000105 | 8.66E-06 | 0.05527 |
| *Gemmiger* | 0.000255 | 3.07E-05 | 0.015563 |
| *Chryseobacterium* | 0.000349 | 0.000261 | 0.743361 |
| *Clostridium* | 0.000221 | 0.000329 | 0.624429 |
| *Rikenella* | 0 | 0.000626 | 0.06829 |
| *Pseudomonas* | 0.001868 | 0.000415 | 0.047988 |
| *Butyricicoccus* | 5.72E-05 | 0.000418 | 0.197725 |
| *Elizabethkingia* | 5.8E-06 | 3.32E-05 | 0.16368 |
| *Ralstonia* | 8.1E-06 | 0.000156 | 0.043033 |
| *Herbaspirillum* | 2.29E-05 | 0.000141 | 0.059257 |
| *Shewanella* | 0.001435 | 0.00031 | 0.146165 |
| *Cronobacter* | 0.000269 | 2.41E-06 | 0.302979 |
| *Sutterella* | 0 | 0.00011 | 0.043599 |
| *Candidatus_Arthromitus* | 0 | 0.000138 | 0.03225 |
| *Dorea* | 0.000113 | 1.57E-06 | 0.014461 |
| *Dehalobacterium* | 0 | 0.000535 | 0.050669 |
| *Collinsella* | 9.63E-05 | 1.75E-06 | 0.031167 |
| *Azomonas* | 0 | 0 | #DIV/0! |
| *Bifidobacterium* | 8.26E-05 | 0.000296 | 0.212805 |
| *Novispirillum* | 0 | 0.000549 | 0.039472 |
| *Subdoligranulum* | 1.16E-05 | 0 | 0.078867 |
| *Comamonas* | 1.18E-05 | 4.66E-05 | 0.275065 |
| *Coprobacillus* | 7.78E-06 | 3.35E-05 | 0.362548 |
| *Azospirillum* | 3.5E-06 | 0.000123 | 0.058936 |
| *Megamonas* | 6.05E-06 | 0 | 0.324375 |
| *Faecalibacterium* | 0.000172 | 1.55E-05 | 0.022382 |
| *p-75-a5* | 6.72E-06 | 1E-05 | 0.738774 |
| *Morganella* | 0.001142 | 3.08E-06 | 0.325667 |
| *Allobaculum* | 0 | 0.000127 | 0.039871 |
| *Bulleidia* | 5.6E-05 | 3.82E-06 | 0.046736 |
